# Extracellular freezing induces a permeability transition in the inner membrane of muscle mitochondria of freeze-sensitive but not freeze-tolerant *Chymomyza costata* larvae

**DOI:** 10.3389/fphys.2024.1358190

**Published:** 2024-02-07

**Authors:** Tomáš Štětina, Vladimír Koštál

**Affiliations:** Institute of Entomology, Biology Centre of the Czech Academy of Sciences, České Budějovice, Czechia

**Keywords:** insects, freeze tolerance, mitochondrial swelling, permeability transition, respiration, oxidative phosphorylation

## Abstract

**Background:** Many insect species have evolved the ability to survive extracellular freezing. The search for the underlying principles of their natural freeze tolerance remains hampered by our poor understanding of the mechanistic nature of freezing damage itself.

**Objectives:** Here, in search of potential primary cellular targets of freezing damage, we compared mitochondrial responses (changes in morphology and physical integrity, respiratory chain protein functionality, and mitochondrial inner membrane (IMM) permeability) in freeze-sensitive vs. freeze-tolerant phenotypes of the larvae of the drosophilid fly, *Chymomyza costata*.

**Methods:** Larvae were exposed to freezing stress at −30°C for 1 h, which is invariably lethal for the freeze-sensitive phenotype but readily survived by the freeze-tolerant phenotype. Immediately after melting, the metabolic activity of muscle cells was assessed by the Alamar Blue assay, the morphology of muscle mitochondria was examined by transmission electron microscopy, and the functionality of the oxidative phosphorylation system was measured by Oxygraph-2K microrespirometry.

**Results:** The muscle mitochondria of freeze-tolerant phenotype larvae remained morphologically and functionally intact after freezing stress. In contrast, most mitochondria of the freeze-sensitive phenotype were swollen, their matrix was diluted and enlarged in volume, and the structure of the IMM cristae was lost. Despite this morphological damage, the electron transfer chain proteins remained partially functional in lethally frozen larvae, still exhibiting strong responses to specific respiratory substrates and transferring electrons to oxygen. However, the coupling of electron transfer to ATP synthesis was severely impaired. Based on these results, we formulated a hypothesis linking the observed mitochondrial swelling to a sudden loss of barrier function of the IMM.

## Introduction

Freezing of body water is lethal for most organisms, but some plants and ectotherms have evolved the ability to survive internal ice formation (i.e., freeze tolerance) (Storey and Storey, 1988; Pearce, 2001). Freeze tolerance is particularly widespread in insects (Asahina, 1970; Sinclair, 1999; Lee, 2010). Ice forms extracellularly in freeze-tolerant organisms, whereas ice formation within their cells is considered lethal (for exceptions, see (Sinclair and Renault, 2010)). Extracellular freezing is an extraordinarily challenging environmental stressor because it combines the deleterious effects of cold, loss of liquid water, elevated osmolality, increased concentrations of protons, calcium, metal ions, and other toxic compounds, anoxia and ischemia, cell shrinkage, mechanical stress, and increased packing of cellular components (Mazur, 1984; Muldrew et al., 2004). The high complexity of freezing stress is an obstacle to experimentation because the hierarchy and importance of individual effects of different stressors on different targets (macromolecules, cellular structures, and processes) are difficult to distinguish. Although considerable knowledge has accumulated over decades, we are still far from an integrative understanding of how cells of naturally freeze-tolerant animals cope with the enormous complexity of freezing stress (Storey and Storey, 2013; Storey and Storey, 2017; Toxopeus and Sinclair, 2018; Teets et al., 2023). Here, we contribute by analyzing the effects of extracellular freezing stress on muscle mitochondrial morphology and function in freeze-sensitive vs. freeze-tolerant phenotypes of naturally freeze-tolerant insects.

Our understanding of how mitochondria in temperate and polar insects adapt to the winter season is still limited (Lubawy et al., 2022; Lebenzon et al., 2023). Overwintering insects are exposed to low ambient temperatures and often spend the winter in diapause—a state of developmental arrest, behavioral inactivity, and deep metabolic suppression (Koštál, 2006; Teets et al., 2023). The relatively low demand for energy turnover, on the one hand, and the high cost of maintaining active mitochondria, on the other hand, led to the evolution of seasonal mitochondrial degradation as an ultimate strategy in some insects. However, such a strategy appears to be exceptional in insects that predictably spend many months of each winter in deep dormancy, such as caterpillars of the moth *Gynaephora groenlandica* that overwinter near the permafrost in Greenland (Kukal et al., 1989; Levin et al., 2003). Some insects, such as the Colorado potato beetle, *Leptinotarsa decemlineata*, at least degrade populations of mitochondria in their seasonally histolyzed flight muscles because the wings are useless in winter (Lebenzon et al., 2022). However, most overwintering insects maintain fully coupled mitochondria capable of oxidizing various substrates throughout the winter. In such insects: 1) the activities of some mitochondrial enzymes may be slightly reduced, as in diapausing larvae of the gall fly *Eurosta solidaginis* and the gall moth *Epiblema scudderiana*, (Ballantyne and Storey, 1985; Joanisse and Storey, 1994; McMullen and Storey, 2008); 2) the relative utilization of different substrates by mitochondrial respiratory complexes may shift, as in cold-acclimated drosophilid flies (Jørgensen et al., 2023); and/or 3) the expression of transcripts encoding the subunits of respiratory complexes may be reduced, as in the cricket *Gryllus veletis* (Toxopeus et al., 2019). Working with the drosophilid fly, *Chymomyza costata*, we showed that winter phenotype larvae (diapausing, cold acclimated) also maintain a high number of fully functional mitochondria in their fat body cells and can effectively protect them from all the deleterious effects associated with extracellular freezing and cryopreservation in liquid nitrogen (Rozsypal et al., 2018; Štětina et al., 2020).

Mitochondria are organelles that remarkably integrate life-sustaining functions and death-promoting activities in eukaryotic cells. Mitochondria are central to cellular energy supply and metabolic transformations, but they also sense changes in the cellular environment and can respond to various physiological imbalances or environmental stressors by inducing cell death pathways (Newmeyer and Ferguson-Miller, 2003; Nunnari and Suomalainen, 2012; Abate et al., 2020; Modesti et al., 2021). It has been hypothesized that the maintenance of mitochondrial integrity and function, while avoiding the induction of death pathways, may be critical for cellular homeostasis and consequently for the survival of the whole organism in ectothermic animals exposed to thermal extremes (Camus et al., 2017; Sokolova, 2018; Chung and Schulte, 2020; Lubawy et al., 2022). Indeed, the experimental evidence available for insects shows that mitochondrial function and integrity are critically affected by cold and freezing stress. For example, data from the vinegar fly *Drosophila melanogaster* show that mitochondrial failure to produce ATP coincides with increased mortality in warm-acclimated flies exposed to chronic cold stress at 4°C for 3 days. Cold-acclimated flies were able to avert both mitochondrial failure and mortality (Colinet et al., 2017). When adult tropical cockroaches *Gromphadorina cocquereliana* were exposed to acute cold shock at 4°C for 3 h, significant changes in respiratory activity, ROS production, and activation of defense systems were detected in their muscle and fat body mitochondria (Chowański et al., 2017). When cockroaches were repeatedly exposed to the same stress for 3 days, an acclimation response was observed as the perturbed mitochondrial parameters returned to homeostasis (Lubawy et al., 2023). We have shown that lethal (but not sublethal) freezing stress applied to summer phenotype (active, warm acclimated) larvae of *C. costata* causes significant swelling of fat body and hindgut mitochondria, resulting in partial loss of tissue respiratory capacity (Štětina et al., 2020). Similar mitochondrial swelling was also observed in mitochondria of Malpighian tubules in lethally frozen larvae of the gall fly, *E. solidaginis* (Collins et al., 1997).

Here, we followed up on our previous observations and compared muscle mitochondrial responses to freezing stress in two contrasting seasonal phenotypes of *C. costata* larvae. The relatively freeze-sensitive (LD, active, warm acclimated, summer phenotype) and freeze-tolerant (SDA, diapause, cold acclimated, winter phenotype) larvae were exposed to freezing stress at −30°C for 1 h, which is lethal for LD larvae but readily survived by SDA larvae (see Materials and Methods for further explanation of LD and SDA abbreviations). We sought answers to the following specific questions: Do muscle mitochondria change their morphology and integrity during freezing stress? Does freezing stress affect the functionality of respiratory complexes in the inner mitochondrial membrane (IMM)? Does freezing stress affect the oxygen consumption capacity and the coupling of the oxidative phosphorylation system (OXPHOS)? Are there differences in mitochondrial responses to freezing stress between LD and SDA larvae? Answers to these questions may improve our understanding of the mechanisms of freezing injury in *C. costata* larvae. From a broader perspective, our results can contribute to the search for integrative models [sensu (MacMillan, 2019)] of freezing injury and its prevention/repair in naturally freeze-tolerant insects and ectotherms.

## Materials and methods

### Insects, acclimations, phenotypes, and tissue dissection

A colony of *C. costata*, Sapporo strain (Riihimaa and Kimura, 1988), was reared on artificial diet in MIR 154 incubators (Sanyo Electric, Osaka, Japan) as described previously (Koštál et al., 1998). This study is based on comparative analysis of muscle mitochondrial responses to freezing stress in two phenotype/acclimation variants of *C. costata* larvae. Two phenotype/acclimation variants with contrasting freeze tolerance were generated according to our earlier acclimation protocols (Rozsypal et al., 2018; Grgac et al., 2022; Kučera et al., 2022): 1) active, warm-acclimated (summer phenotype) → freeze-sensitive larvae (abbreviated as LD in earlier papers); 2) diapause, cold-acclimated (winter phenotype) → freeze-tolerant larvae (abbreviated as SDA in earlier papers). Briefly, the LD larvae were reared from eggs to 3rd larval instars (age of 3 weeks) at 18°C under long-day photoperiod (LD, 16 h light:8 h dark), which promotes direct non-diapause development to pupa and adult. The SDA larvae were reared until age of 6 weeks at 18°C under short-day photoperiod (SD, 12 h light:12 h dark)—which induces larval diapause—and were then transferred to constant darkness and progressively cold acclimated over 5 weeks (SDA, 1 week at 11°C, followed by 4 weeks at 4°C). The LD larvae of *C. costata* have limited survival after freezing stress (35% survive slow inoculative extracellular freezing to −5°C, 10% survive to −10°C, and none survive freezing to −20°C or below). In contrast, practically all SDA larvae survive freezing to −30°C or even −75°C, and 42.5% survive and metamorphose into fit adults after 18 months of cryopreservation in liquid nitrogen (Rozsypal et al., 2018).

The larval muscles form a dense three-dimensional system of longitudinal and dorsoventral muscles below the body wall (Wiplfler et al., 2013). We dissected the larvae under binocular microscope and ensured that approximately 90% of the total larval body wall muscle volume was taken from each dissected larva. The larvae were dissected in different buffers for different purposes: 1) for transmission electron microscopy (TEM): in ice-cold phosphate-buffered saline (PBS: 137 mM NaCl, 3 mM KCl, 10.1 mM Na_2_HPO_4_, and 1.8 mM KH_2_PO_4_, pH 7.2); 2) for citrate synthase activity assay: in Tris-sucrose buffer (50 mM Tris-HCl, 200 mM sucrose, 1 mM EDTA, 0.1% Triton-X 100, pH 8.0); 3) for Alamar Blue assays and for Oxygraph-2K respirometry: in Schneider’s insect medium (Biosera, Nuaillé, France). The dissection of single larva took less than 1 min.

### Freezing stress

Whole larvae were exposed to slow inoculative freezing to −30°C using the protocol described earlier (Rozsypal et al., 2018). The protocol consisted of five steps set in a Ministat 240 programmable cryostat (Huber, Offenburg, Germany): 1) 20 min of larval manipulation at 0°C (washing larvae out of the diet, dividing into groups of 20 specimens, and placing them into 3 mL plastic tubes between two layers of moist cellulose); 2) slow freezing to −30°C (with a small ice crystal added on top of the moist cellulose) for 300 min (cooling rate, 0.1°C min^−1^); 3) keeping larvae at −30°C for 60 min; 4) heating from −30°C to +5°C over 60 min (heating rate, 0.6°C min^−1^); and 5) keeping at constant +5°C for 10 min to allow complete melting of ice. Adding a small ice crystal at the beginning of step (2) induces inoculative internal freezing in larvae, which starts between −1°C and −3°C and results in freezing of 76% or 68% of total body in LD or SDA larvae, respectively (Rozsypal et al., 2018). This freezing stress is invariably lethal for LD larvae while it is survived by SDA larvae in rates similar to unfrozen controls.

### Transmission electron microscopy

The TEM was conducted as described earlier (Des Marteaux et al., 2018; Štětina et al., 2020). Briefly, the larval muscles were dissected in PBS right upon melting—at the end of step (5) of the freezing protocol (control larvae were not frozen). The muscles were fixed overnight in PBS containing 2.5% glutaraldehyde. After washing in PBS, we continued in fixation using 2% OsO_4_ (EMS, Hatfield, Pennsylvania) for 2 h, washing in PBS, dehydration in grading acetone solutions (from 30% to 100%), and embedding into resin Embed-812 (EMS). The resin was polymerized at 60°C/24 h and the blocks were sectioned using an ultra-microtome Leica UC6 (Leica microsystems GmbH, Wetzlar, Germany) equipped with DiATOME diamond knife (EMS). The ultra-thin sections (70 nm) were placed on 300-mesh-Cu grids and counter-stained with saturated ethanolic uranyl acetate for 30 min, followed by lead citrate for 20 min. The grids with sections were then coated by carbon film using JEOL JE 4C coater (JEOL, Tokio, Japan), and micrographs were taken by a transmission electron microscope JEOL JEM—1010 1 (JEOL, Tokio, Japan).

### Morphology of muscle mitochondria

Based on our earlier study, we focused on mitochondrial swelling as this was the most characteristic response to freezing stress in fat body and hindgut mitochondria of *C. cos*tata larvae (Štětina et al., 2020). We distinguished three morphological categories 1) normal size, shape, and presence of cristae; 2) transitional (slightly enlarged, rounded, cristae invisible, matrix still containing the electron-dense material); and 3) swollen (large, rounded, OMM sometimes discontinuous—bursting, internal structure lost, matrix showing little or no electron dense material). The examples of mitochondrial morphology scoring are shown in Supplementary Figure S1.

Muscles dissected from five larvae (each larva considered as a biological replicate) of each combination of phenotype/acclimation variant and freezing treatment were collected for TEM analysis. We selected approximately 20 representative TEM micrographs (magnification of ×25,000), taken at different locations of the ultra-thin section, in order to collect morphology scores for several hundred mitochondria in every single larva (the exact numbers are shown in Supplementary Tables). In total, we scored the morphology of 6,291 muscle mitochondria. A gallery containing all micrographs and scores is available in figshare: https://figshare.com/articles/figure/Gallery_of_mitochondria_pdf/24961422.

### Citrate synthase activity

Muscle tissues dissected from 3 × 10 larvae (3 biological replicates, 10 larvae pooled for each replicate) of each combination of phenotype/acclimation variant and freezing treatment were collected for citrate synthase activity assay. The tissue samples were homogenized in 600 µL of the Tris-sucrose buffer using metal-blade homogenizer X 120 (Ingenieurbüro CAT M. Zipperer, Germany) followed by sonication using 4710 Series Ultrasonic Homogenizer (Cole Parmer, Chicago, IL, United States). After the incubation of homogenate for 10 min at 0°C, the samples were centrifuged at 22,000 *g*/10 min/4°C, and the supernatants were used as the source of enzyme. All activity assays were run at constant 25°C. The composition of standard reaction mixture for CS assay was similar as described earlier (Štětina et al., 2020; Grgac et al., 2022): 20 mM imidazole-HCl buffer, pH 7.2; 0.05 mM DTNB reagent (5,5′-dithiobis-2-nitrobenzoic acid); 0.15 mM acetyl CoA; 0.25 mM oxaloacetate. The reaction was initiated by the addition of 25 μL of oxaloacetate solution into 475 μL of reaction mixture and the mercaptid ion formation from CoA-SH (that was released upon synthesis of citrate) was followed by measuring the increase of 412 nm absorbance (DTNB → TNB). The total proteins were measured by bicinchoninic acid assay according to Smith et al. (1985).

### Alamar Blue assay of metabolic activity of muscle cells

The alamarBlue™ HS (Invitrogen, Thermo Fisher Scientific, Waltham, Massachusetts, United States) is a cell-viability assay based on a principle that the innate metabolic activity of living cells maintain reducing conditions that result in a chemical reduction of resazurin to resorufin (Alamar Blue reagent), which is accompanied with the change of color that can be measured spectrometrically (Supplementary Figure S2B). The oxidation-reduction potential of the Alamar Blue reagent is intermediate between the final reduction of O_2_ and cytochrome *c* oxidase, and Alamar Blue can thus be used as a proxy metabolic indicator of the mitochondrial electron transfer chain activity (Braut-Boucher and Aubery, 2017). However, in addition to mitochondrial reductases, the Alamar Blue reagent is reduced by several other enzymes (Rampersad, 2012), so we conservatively interpret the Alamar Blue reagent reduction as an indicator of cellular metabolic activity.

Muscle tissues dissected from 3 × 5 larvae (3 biological replicates, 5 larvae pooled for each replicate) of each combination of phenotype/acclimation variant and freezing treatment were collected for metabolic activity assay. The tissues were transferred into 200 µL of Schneider’s medium in the wells of 96-well EIA/RIA polystyrene plate (Corning, NY, United States). The 10 µL of Alamar Blue reagent was added into each well, mixed, and the plates were incubated at constant 18°C for 6 hours. The metabolic activity was inferred based on gradual color change of the assay from blue to pink. The change of color was recorded by photographing using cell phone and standardized lighting conditions in dark chamber. At the end of assay, at a time of 360 min, the absorbances at 600 nm and 570 nm (resazurin and resorufin, respectively) were taken. In addition, we ran Alamar Blue assays in: 1) blanks (assays without tissue samples); and 2) muscles of LD larvae exposed to heat shocks of 45°C or 65°C for 1 h (both heat shocks are invariably lethal for LD larvae).

### Oxygraph-2K respirometry of the OXPHOS complexes I and II

Muscle tissues dissected from 3 × 20 larvae (3 biological replicates, 20 larvae pooled for each replicate, which corresponds to approximately 1–2 mg of tissue in each replicate) of each combination of phenotype/acclimation variant and freezing treatment were collected for microrespirometry using the Oxygraph-2K respirometer (Oroboros Instruments, Innsbruck, Austria). We measured oxygen consumption in permeabilized muscle tissue, which preserves intact mitochondria well integrated into the cellular system and, therefore, provides physiologically relevant insight into mitochondrial function (Kuznetsov et al., 2008). Our experimental approaches were designed basically according to principles described earlier (Pichaud et al., 2013; McDonald et al., 2018; Simard et al., 2018). The dissected muscles were washed 3 times in 1 mL of respiration buffer (115 mM KCl, 10 mM KH_2_PO_4_, 2 mM MgCl_2_, 3 mM HEPES, 1 mM EGTA, 0.2% BSA, pH 7.2) and then transferred to 2 mL of respiration buffer in the Oxygraph-2K chamber. All microrespirometry assays were run at constant 25°C. The tissue was permeabilized by 55 µM or 30 µM digitonin for LD and SDA acclimation variants, respectively (see Supplementary Figure S2C for explanation) and the oxygen consumption rates representing the basal respiration prior to adding substrates was taken (this respiration was likely supported by substrates present endogenously in dissected muscle cells). Then, the specific substrates for complexes I and II were added, which stimulated the mitochondrial oxygen flux. In the absence of ADP, this flux mainly represents the compensation of the proton leak (i.e., LEAK state respiration rate). However, since we cannot exclude the possibility that some tissue-endogenous ADP contributed to the oxygen consumption rate, we will refer to this oxygen flux as the substrate-stimulated (SS) oxygen flux. Finally, exogenous ADP was added to further stimulate oxygen consumption and the OXPHOS state respiration rate was estimated (the mitochondrial oxygen flux corresponding to electron transfer chain coupled to phosphorylation and ATP production by complex V, ATP synthase).

The oxygen consumption rates after each addition were estimated when the signal stabilized and were expressed as pmol O_2_/(s.10 muscles)^−1^. It was technically difficult to either weight the fresh mass of larval muscle tissue prior to analysis, or quantitatively recover the muscle tissue from respiration vials after the analysis (to measure total protein). Therefore, we normalized the data to 10 muscle tissues which corresponds directly to the reading of Oxygraph-2K [O_2_/(s.mL)^−1^], where the total volume is 2 mL and 20 tissues were pooled. We believe that this normalization is sufficient for comparative purposes. The order of additions was as follows (the concentrations in brackets are the final concentrations in respiration buffer): complex I, 1) the substrates pyruvate (10 mM) and malate (2 mM); 2) the ADP (2.5 mM) to couple the OXPHOS; and rotenone (1 μg.m^−1^) to inhibit the activity of complex I. Complex II, 1) rotenone (1 μg.m^−1^) to block the recurrent electron flow to complex I; 2) the substrate succinate (10 mM); 3) ADP (2.5 mM); and 4) KCN (5 mM) to inhibit the whole electron transfer chain by blocking the Complex IV (cytochrome *c* oxidase). The basal, SS, and OXPHOS oxygen consumption rates were used to calculate two parameters separately for complexes I and II: 1) the substrate contribution ratio, SCR = (SS—basal)/basal; and 2) the OXPHOS coupling efficiency = 1—(SS/OXPHOS) (Menail et al., 2022). See Supplementary Figure S2A for schematic depiction of our experimental approaches to the analysis of activity of different complexes of the OXPHOS residing in the inner mitochondrial membrane.

### Statistics

Statistical analyses were performed with generalized linear models (GLM) and with linear ANOVA models followed by Tukey’s *post hoc* multiple comparison tests when the ANOVA was significant. To respect the assumptions of parametric ANOVA model, the normality of model residuals was checked with Shapiro-Wilk test and the homoscedasticity with the Bartlett test. When the assumptions of parametric model were not met, data were log-transformed. If neither log-transformation have satisfied the assumptions of parametric model, we used the test with rank-transformed response variables. All analyses were performed with the R software version 4.3.2 (R Core Team, 2023), multiple comparisons with the multcomp package (Hothorn et al., 2008). We provide the raw data used for statistical analyses in the Supplementary Tables.

## Results

### Muscle mitochondria are morphologically similar in LD and SDA control larvae

**FIGURE 1:**
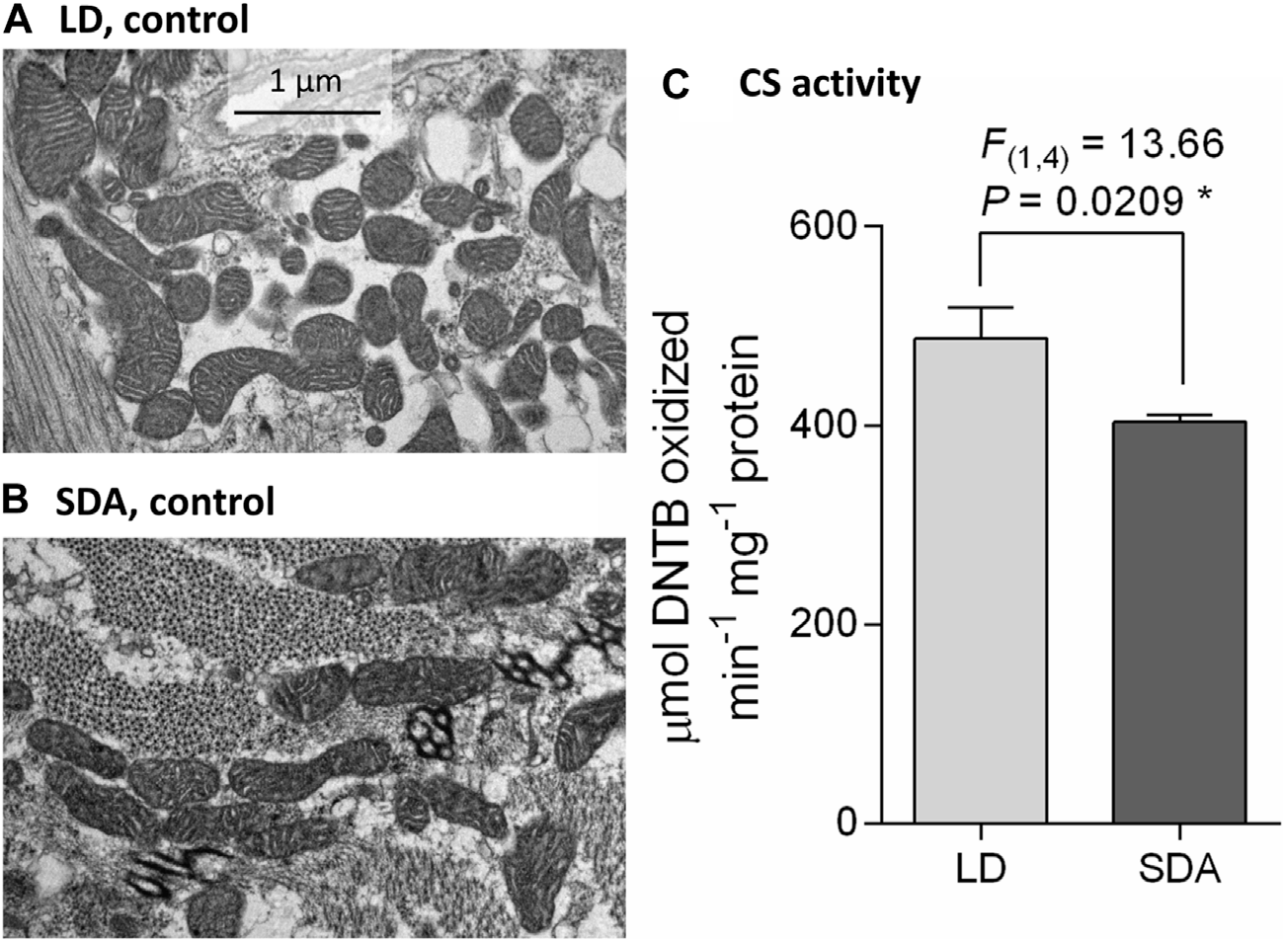
Larval muscle mitochondria are morphologically similar in two acclimation variants of *Chymomyza costata*. The transmission electron micrographs taken at a magnification of ×25,000 show examples of typical muscle mitochondria in control (unfrozen) larvae of two phenotypes: **(A)** LD, freeze sensitive; and **(B)** SDA, freeze tolerant. More examples are presented in Supplementary Figure S3, and a gallery containing all micrographs is available in figshare. **(C)** Mean citrate synthase (CS) activity in SDA muscles was 83% of that in LD muscles. Each column shows mean ± SD (*n* = 3, each replicate is a pool of 10 dissected larval muscle tissues). The difference between LD and SDA larvae was analyzed using ANOVA model (*p < 0.05, significantly different).

Mitochondria in the larval muscles of control (unfrozen) larvae were of broadly similar morphology (Figures 1A, B), although the shape of individual mitochondria was highly variable, ranging from spherical to rod-shaped and elongated (see Supplementary Figure S3 for diversity of mitochondrial morphology; a gallery containing all micrographs and scores is available in figshare). On transverse section, the diameter of individual mitochondria did not exceed 1 µm and their length did not exceed 3 µm. The cristae of the inner membrane were well developed. Such mitochondria were classified as “normal” (category 1) (Supplementary Figure S1). There was no apparent difference in mitochondrial number between acclimation variants LD and SDA. However, the citrate synthase activity normalized to total protein (a proxy for mitochondrial number) in SDA larval muscles was only 83% of that in LD larval muscles (Figure 1C).

### LD mitochondria swell while SDA mitochondria remain intact after freezing stress

**FIGURE 2:**
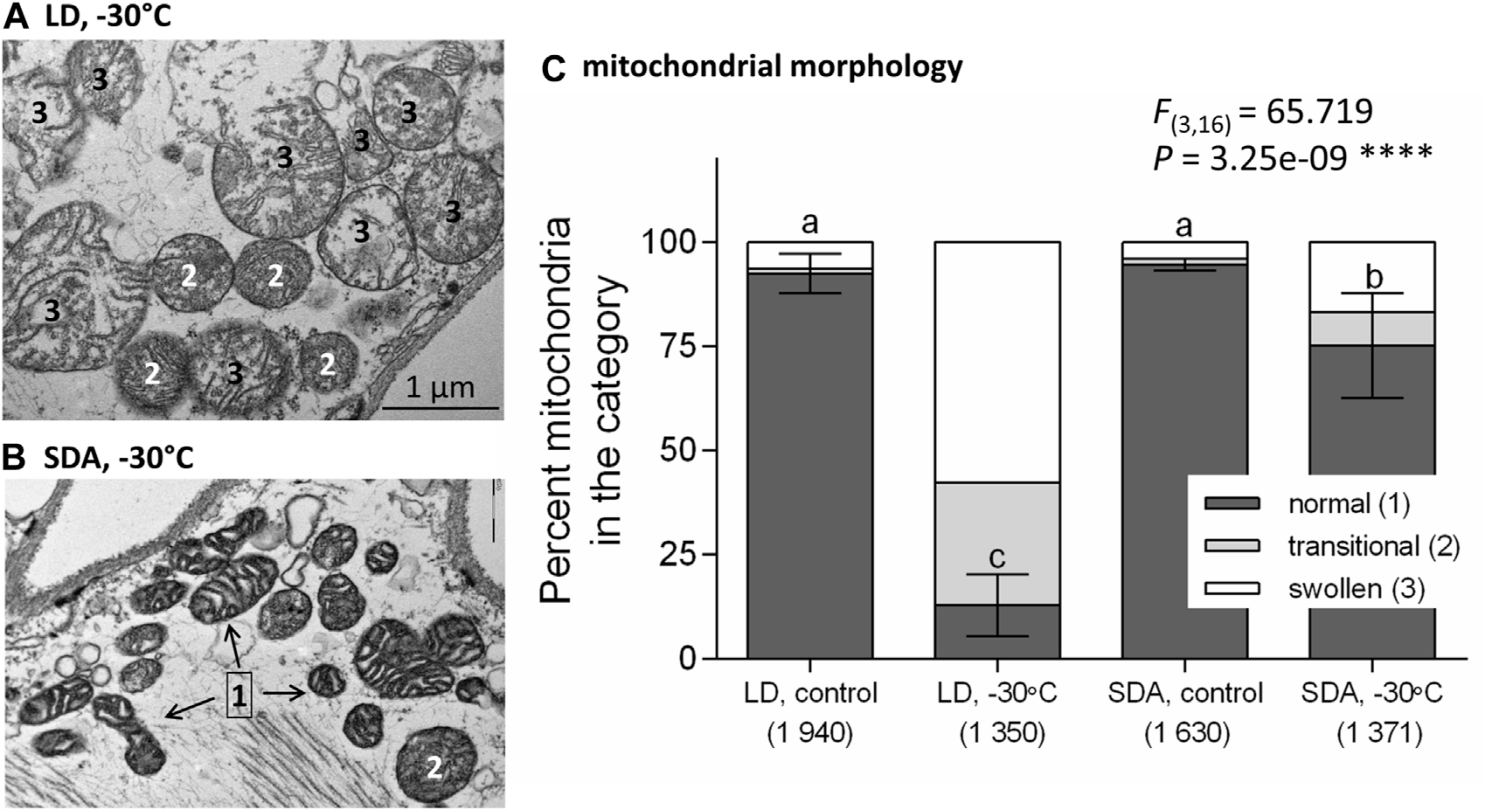
Morphological responses of larval muscle mitochondria to freezing stress differed in two acclimation variants of *Chymomyza costata*. The larvae were exposed to slow inoculative freezing to −30°C, which is lethal to freeze-sensitive LD larvae but survived by freeze-tolerant SDA larvae. The muscle tissues were dissected from five larvae of each experimental variant: a combination of acclimation, LD vs. SDA, and treatment. The transmission electron micrographs taken at a magnification of ×25,000 show examples of **(A)** swollen mitochondria in LD larvae in contrast to **(B)** morphologically normal mitochondria of SDA larvae. More examples are presented in Supplementary Figure S4, and a gallery containing all micrographs is available in figshare. **(C)** The relative proportions of mitochondria with morphology classified as normal (1), transitional (2), and swollen (3) (see Supplementary Figure S1 for a classification scale). Each stacked column shows mean proportions of three morphological classes. The variation (±SD) is shown for “normal” class only (*n* = 5, single larva was taken as an independent replicate, and total numbers of mitochondria classified in each experimental variant are shown in parentheses). Statistical analysis using ANOVA model followed by Tukey’s multiple comparisons test was performed for proportions of “normal” mitochondria only, as the proportion of “damaged” mitochondria (transitional + swollen) is only a complement to 100%. Stars represent statistical differences (****p < 0.0001). Columns flanked by different letters are statistically different.

Upon freezing stress, most LD mitochondria responded with a characteristic swelling (Figure 2A, see also Supplementary Figure S4A for more examples of swollen mitochondria). The shape became predominantly rounded, the diameter increased to more than 1 µm. The “empty” spaces (loss of electron-dense material) occurred in the mitochondrial matrix, and the structure of the cristae was partially or completely lost. Some mitochondria had discontinuous outer membrane and looked like burst. Such mitochondria were classified as transitional (2) or swollen (3) (Supplementary Figure S1).

In contrast, many SDA mitochondria retained their normal morphology after freezing stress and were classified as category (1) (Figure 2B; Supplementary Figure S4B). Statistical analysis revealed highly significant effect of freezing stress on morphology of mitochondria. While the relative proportion of damaged morphologies (transitional + swollen) increased with freezing stress, the relative proportion of normal morphologies decreased from 92.4% before stress to 12.6% after stress in LD mitochondria, while it decreased much less, from 94.8% to 72.7%, in SDA mitochondria (Figure 2C).

### Muscle mitochondria are functionally similar in LD and SDA larvae but respond differently to freezing stress

**FIGURE 3:**
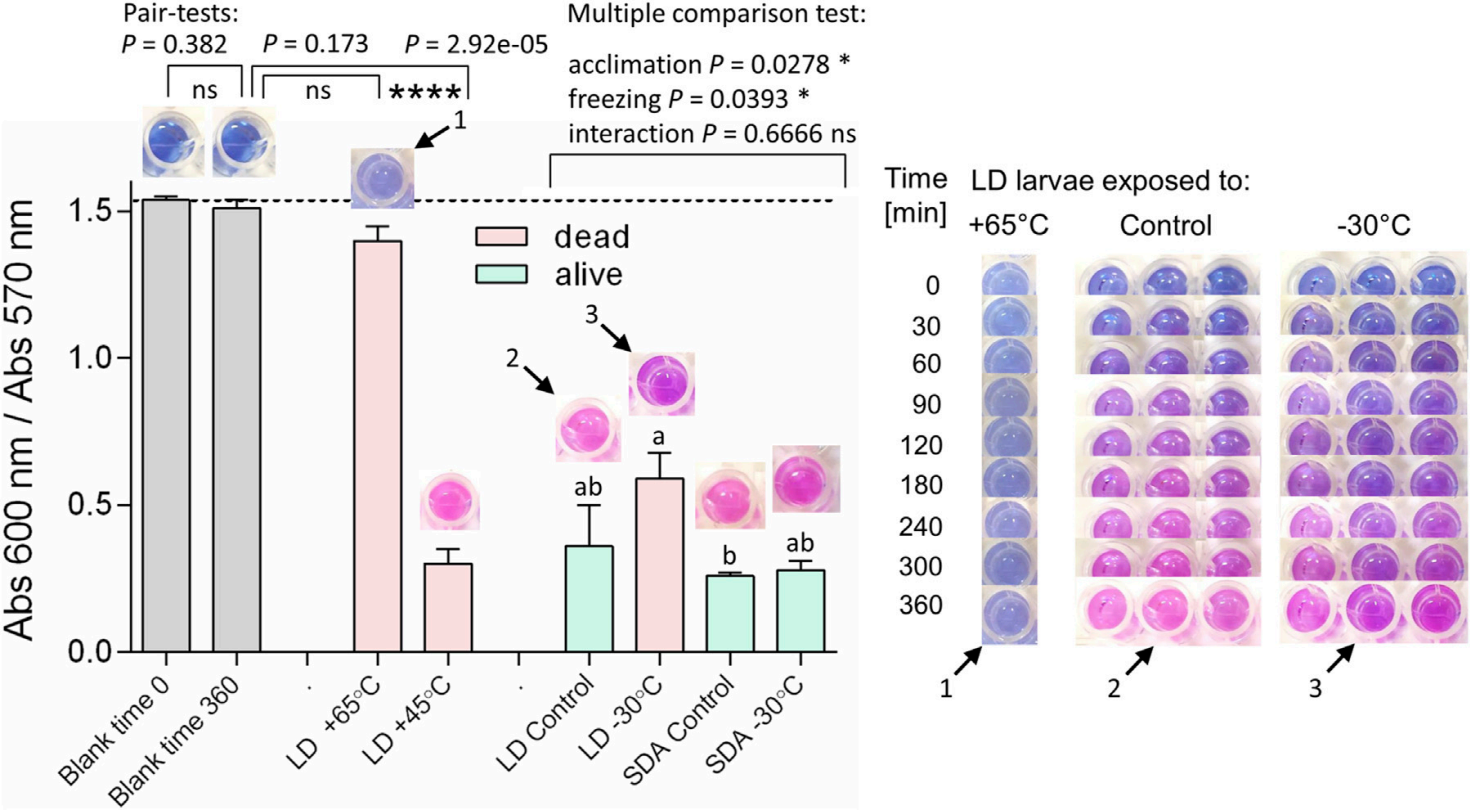
The metabolic activity persisted in larval muscle cells of *Chymomyza costata* after the lethal freezing stress. Each column shows mean ± SD (*n* = 3, each replicate is a pool of five dissected larval muscle tissues) ratio of Alamar Blue assay absorbance at 600/570 nm taken at the incubation time of 360 min (see text and Supplementary Figure S2B for explanations). Abbreviations: LD, freeze-sensitive larvae, SDA, freeze-tolerant larvae. The photographs above each column show examples of the color of Alamar Blue assay after 360 min. The set of photographs on the right show examples of gradual change in color of the Alamar Blue assay from blue (600 nm) to pink (570 nm) over the incubation time in select treatments (single replicate of the LD larvae exposed to heat shock at 65°C; all three replicates of LD control; all three replicates of LD frozen at −30°C). No color change with time of incubation (meaning no metabolic activity) was observed in blank assays (no tissue); minimum change in color was also seen in the assays containing muscle tissue dissected from larvae killed by heating to 65°C; the larvae killed by heating to 45°C, however, still showed a change in color. Similarly, the freeze-sensitive LD larvae killed by freezing stress still showed a significant change in color (meaning some metabolic activity), though this change was slightly slower than the change in color in the assays of control (unfrozen) LD or SDA larvae or frozen SDA larvae (which readily survive the freezing stress). The differences between blanks and heat-stressed LD larvae were statistically analyzed using ANOVA models (see clamps for compared pairs). In order to apply two-way ANOVA model followed by Tukey’s multiple comparisons to analyze differences among control and frozen larvae, the rank-transformed response variables were used. Stars represent statistical differences (*p < 0.05; ****p < 0.001; ns, not significant). Columns flanked by different letters are statistically different.

The results of the Alamar Blue assays, summarized in Figure 3, suggest that the innate metabolic activity of LD muscle cells persists, at least in part, after the organism is killed by freezing or even heat stress. No or little metabolic activity (i.e., no or little change in the color of the assay over 6 h) was observed only in the blanks (no tissue in the assay) and in the muscles of larvae killed by heating to 65°C. Muscles from LD larvae killed by either heating to 45°C or freezing to −30°C still showed clear metabolic activity, as indicated by a change in the color of the assay from blue to pink. Calculation of the ratio of light absorbance at 600/670 nm at 360 min allowed relative quantification of the color change and suggested that the metabolic activity was significantly reduced (but not arrested) by freezing stress in LD muscles compared to either control LD muscles or control and frozen SDA muscles (SDA larvae readily survive freezing stress).

**FIGURE 4:**
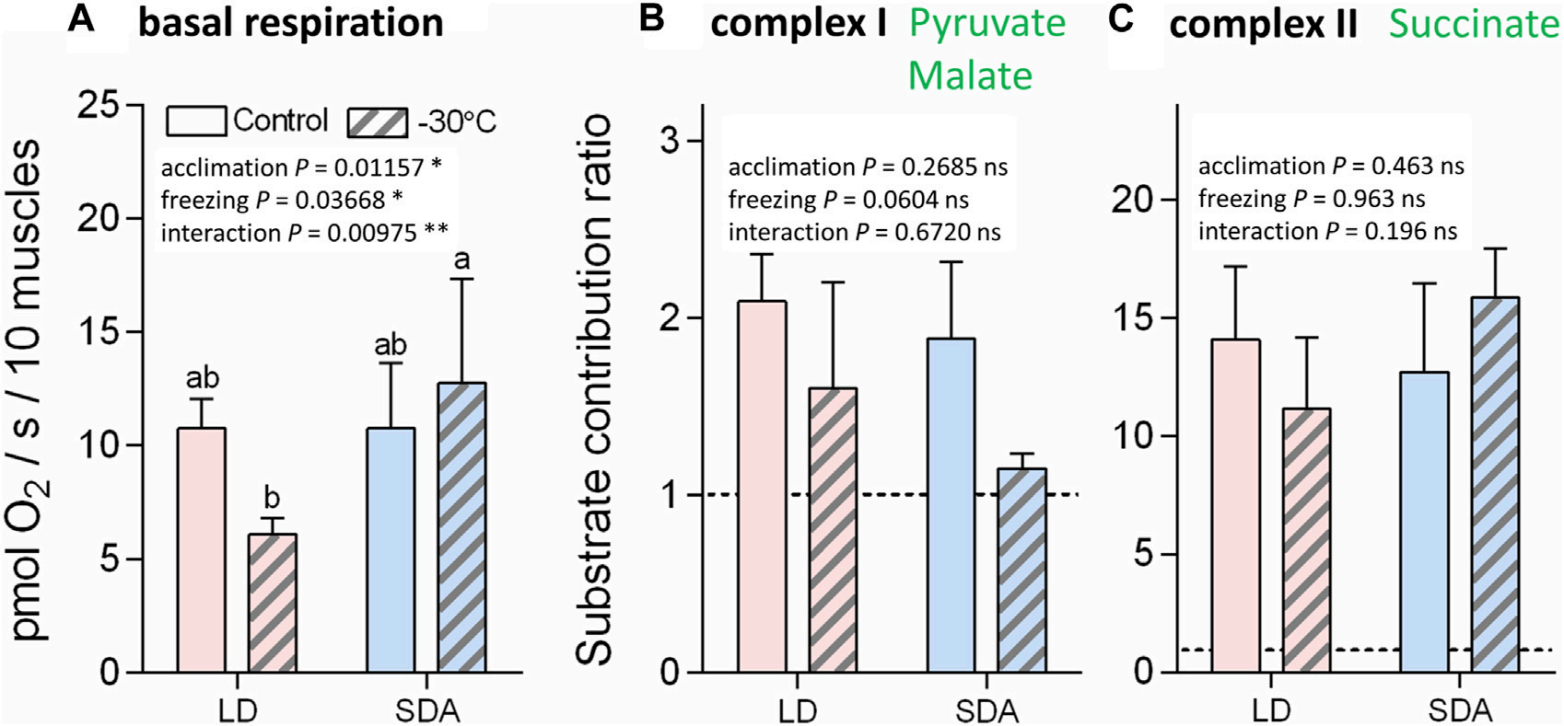
The basal respiration rates and substrate contribution ratios of larval muscle mitochondria prior to and after freezing stress in two acclimation variants of *Chymomyza costata*. The freeze-sensitive (LD) and freeze-tolerant (SDA) larvae were exposed to slow inoculative freezing to −30°C. The muscle tissues were dissected right after the freezing stress (or taken from control-unfrozen larvae), permeabilized using digitonin, and the oxygen consumption rates were measured using Oxygraph-2K respirometer prior to addition of any substrates to measure **(A)** the basal respiration. Next, substrates for complex I **(B)** and complex II **(C)** were added and the corresponding increase of oxygen consumption rate was expressed as substrate contribution ratio (SCR, see text for explanation). Each column shows mean ± SD [*n* = 6 in **(A)** or *n* = 3 in **(B,C)**, each replicate is a pool of 20 dissected larval muscle tissues]. The differences were statistically analyzed using two-way ANOVA model followed by Tukeys’s multiple comparisons test. Stars represent statistical differences (*p < 0.05; **p < 0.01; ns, not significant). Columns flanked by different letters are statistically different. In case of basal respiration **(A)**, the rank-transformed response variables were used.

The basal respiration rates of digitonin-permeabilized muscle tissue prior to addition of substrates for complexes I or II were practically equal in the LD and SDA control muscles [10.8 pmol O_2_/(s.10 muscles)^−1^] (Figure 4A). In response to freezing stress, the basal respiration rate decreased to 6.1 pmol O_2_/(s.10 muscles)^−1^ in the LD muscles, while it marginally increased to 12.8 pmol O_2_/(s.10 muscles)^−1^ in the SDA muscles (none of these changes, however, was statistically significant). The substrate contribution ratios were much lower for pyruvate and malate (ranging from 1.1 to 2.1, Figure 4B) than for succinate (ranging from 11.1 to 15.1, Figure 4C). Statistical analysis revealed no significant effects of larval acclimation (LD vs. SDA) or freezing (control vs. −30°C) on the response of complexes I and II to their substrates.

**FIGURE 5:**
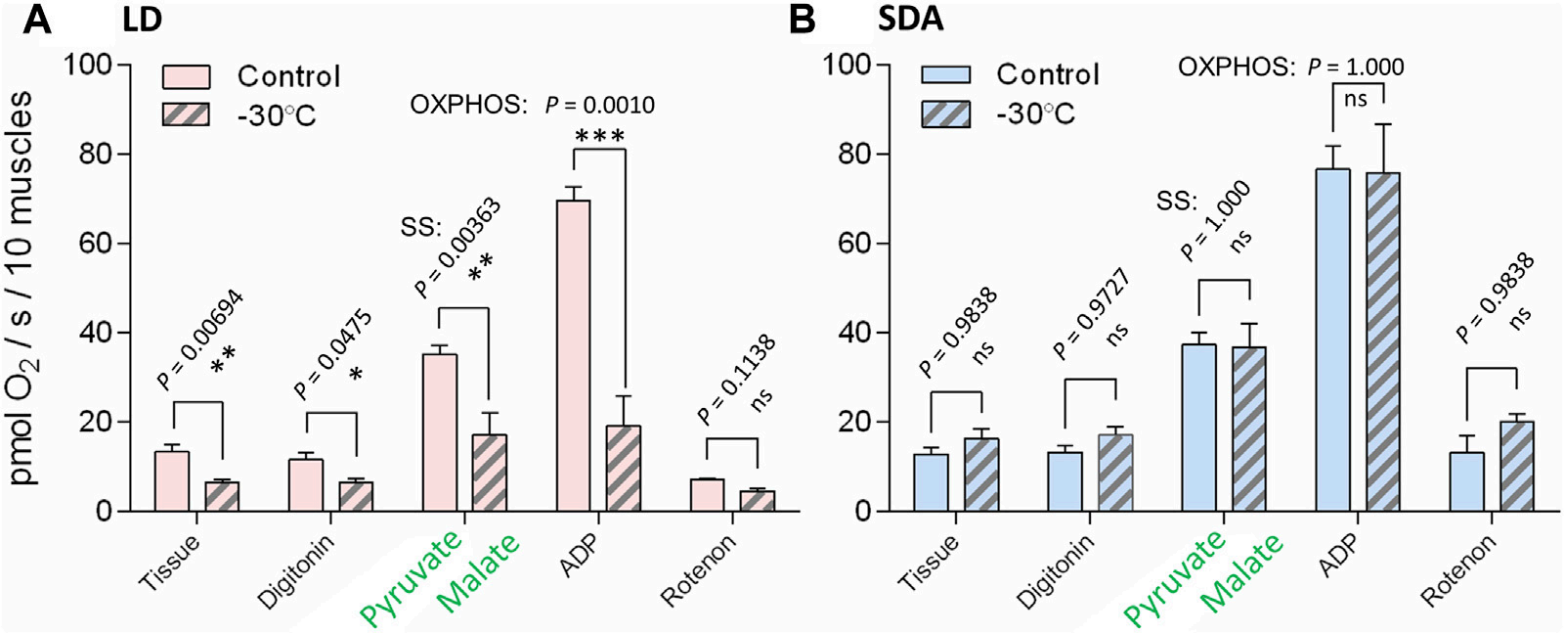
The respiration rates linked to activity of complex I were significantly reduced after the freezing stress in the muscles of freeze-sensitive larvae of *Chymomyza costata*. The freeze-sensitive (**A**, LD) and freeze-tolerant (**B**, SDA) larvae were exposed to slow inoculative freezing to −30°C. The muscle tissues were dissected right after the freezing stress (or taken from control-unfrozen larvae), permeabilized using digitonin, and the oxygen consumption rates were measured using Oxygraph-2K respirometer after adding pyruvate and malate—specific substrates for complex I (substrate-stimulated oxygen flux, SS), followed by ADP (OXPHOS state, activity of electron transfer chain coupled to ATP synthase), followed by rotenone—specific inhibitor of complex I (see Supplementary Figure S2A). Each column shows mean ± SD (*n* = 3, each replicate is a pool of 20 dissected larval muscle tissues). The differences between frozen and control variants (see clamps) were statistically tested using multiple ANOVA models (checked for nested effects). Stars represent statistical differences (*p < 0.05; **p < 0.01; ***p < 0.001; ns, not significant). In case of LD larvae **(A)**, the log-transformed oxygen consumption data were used. See Supplementary Figure S5 for examples of original oxygen flux traces taken by Oxygraph-2K.

**FIGURE 6:**
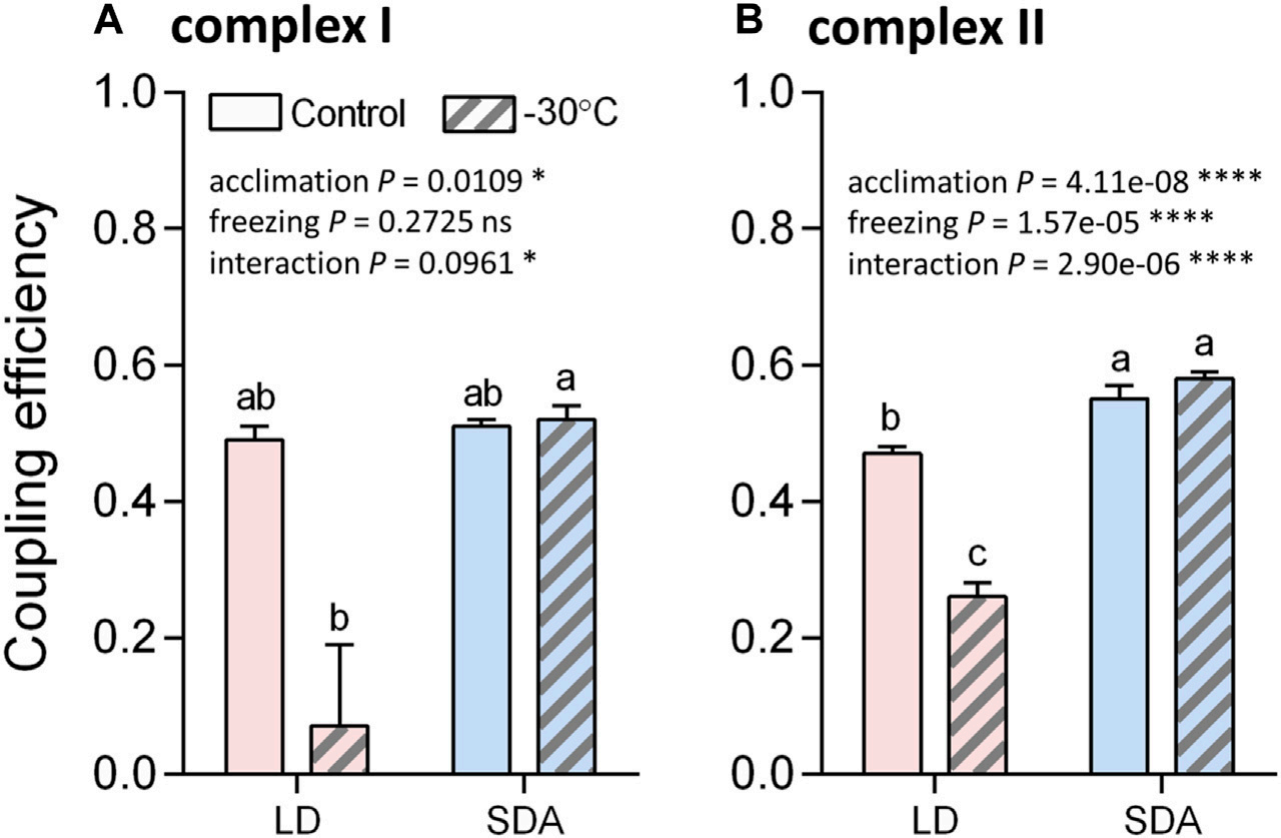
The efficiency of coupling between electron transfer chain and ATP synthase significantly decreased after the freezing stress in the muscles of freeze-sensitive larvae of *Chymomyza costata*. The coupling efficiencies were calculated from oxygen flux data (Figures 5 and Supplementary Figure S6), separately for complexes I **(A)** and II **(B)**, using a formula: efficiency = 1—(SS/OXPHOS). Each column shows mean ± SD (*n* = 3, each replicate is a pool of 20 dissected larval muscle tissues). The differences were statistically analyzed using two-way ANOVA model followed by Tukeys’s multiple comparisons test. Stars represent statistical differences (*p < 0.05; ****p < 0.0001; ns, not significant). Columns flanked by different letters are statistically different. In case of complex I **(A)**, the rank-transformed response variables were used.

Oxygen consumption rates supported by complex I activity (Figure 5, see Supplementary Figure S5 for examples of original Oxygraph-2K traces of oxygen concentration and flux) decreased significantly after freezing stress in LD larvae (Figure 5A). This decrease in oxygen consumption rate was expressed in all steps of the respirometric analysis, from basal rates, to SS respiration energized by the substrates pyruvate and malate, to OXPHOS respiration of ADP-stimulated mitochondria. In contrast, the rates of oxygen consumption associated with complex I activity were virtually unaffected by freezing stress in SDA larvae (Figure 5B). The response of complex II to freezing stress was generally similar to that of complex I, i.e., the rates of oxygen consumption decreased significantly after freezing stress in LD larvae, whereas the rates remained unchanged after freezing stress in SDA larvae (Supplementary Figures S6, S7). The coupling efficiencies calculated for complexes I and II considerably decreased in response to freezing stress in LD muscles, whereas they remained practically unchanged in SDA muscles (Figure 6).

## Discussion

Here we show that switching *C. costata* phenotypes from an active, warm acclimated larva (LD, summer phenotype, freezing sensitive) to a diapausing, cold acclimated larva (SDA, winter phenotype, freezing tolerant) has relatively little effect on muscle mitochondrial numbers, morphology, functionality of OXPHOS complexes, and oxygen consumption (measured at constant 25°C). In contrast, the responses of LD and SDA muscle mitochondria to freezing stress at −30°C were drastically different: while SDA mitochondria remained morphologically and functionally intact, LD mitochondria swelled and became partially dysfunctional. These results confirm and extend our previous observations of similar mitochondrial responses to freezing stress in fat body and hindgut tissues of *C. costata* larvae (Štětina et al., 2020). We will discuss mechanisms that may cause the observed freeze-induced mitochondrial dysfunction and physical disintegration, distinguishing between damage to protein respiratory chain complexes and to the inner mitochondrial membrane (IMM) in which the complexes are located.

### The protein respiratory chain complexes remain partially functional in lethally frozen insects

Our results suggest that the respiratory complexes in *C. costata* muscle mitochondria survive freeze-induced death of the whole organism with only partial loss of activity. The Alamar Blue assay showed that the metabolic activity of muscle cells slows down, but does not stop, in LD larvae after lethal freezing stress. Oxygraph-2K respirometry confirmed this result, showing that the mitochondrial respiration still proceeds in the muscles of lethally frozen LD larvae. The rate of basal oxygen consumption (without added substrates) decreased to 56.6% in lethally stressed LD larvae compared to unfrozen controls. Nevertheless, complexes I and II still clearly responded to their respective substrates in lethally frozen LD larvae. For example, the succinate-stimulated oxygen consumption (i.e., the succinate contribution ratio) was 11.1-fold higher than the basal consumption rate in lethally frozen LD larvae. These results indicate that the entire electron transfer chain from complex I or II via complexes III and IV to oxygen must have functioned at least partially in lethally stressed LD larvae. Thus, the “proteinaceous component” of the mitochondrial respiratory system in *C. costata* larval muscles appeared to be relatively resistant to freezing stress. In addition, the electron transfer chain complexes were found to be relatively stable even under lethal heat stress. The Alamar Blue assay indicated that metabolically active mitochondria survived in muscle cells of *C. costata* larvae killed by heat stress at 45°C. A higher temperature of 65°C was required to completely “kill” the metabolic activity in *C. costata* muscles, probably via irreversible denaturation of enzymes, including the electron transfer chain proteins. Similar results, showing that *in vitro* mitochondrial respiration still proceeds at temperatures above the upper thermal limit for organismal survival, were obtained for adults of the vinegar fly *D. melanogaster* (Heinrich et al., 2017). Taken together, these results suggest that proteins forming the mitochondrial electron transfer chain in *C. costata* muscle do not represent the first line of sensitivity to thermal stress and retain at least partial activity after organismally lethal freezing stress.

Furthermore, we observed that muscle mitochondria from lethally frozen larvae of *C. costata* are decoupled, i.e., they respond to exogenous ADP with a significantly lower increase in oxygen consumption than mitochondria from control or freeze-tolerant larvae exposed to the same stress. Considering that complexes I-IV of lethally frozen larvae remain partially functional, we speculate that the dissociation between proton gradient generation and its use for ATP synthesis by complex V is caused by increased leakage of the IMM for protons or, more generally, by permeabilization of the IMM and loss of its barrier function.

### Lethal freezing stress is associated with permeability transition in IMM

Despite the maintenance of partially functional respiratory complexes I-IV, mitochondria from lethally frozen LD larvae of *C. costata* exhibited morphological changes characteristic of a permeability transition in the IMM: the mitochondrial matrix appeared diluted, the cristae were displaced to the periphery or lost, and the mitochondria were significantly swollen, sometimes showing discontinuous outer membranes (burst). In healthy mitochondria, matrix volume is regulated by constant effluxes of cations (mainly K^+^ and Ca^2+^) across the IMM, which are energized by a steep proton gradient (ΔΨm of about −180 mV) formed by proton pumping activities of complexes I, III and IV of the electron transfer chain (Kaasik et al., 2007; Javadov et al., 2018). When the IMM loses its barrier function, the proton gradient dissipates, which has two major consequences: first, the mitochondria are decoupled, and second, the regulated efflux of cations is impaired. The resulting colloidosmotic gradient caused by accumulated cations and tightly packed proteins in the matrix will then drive water in and cause mitochondrial swelling (Halestrap, 2009; Bernardi et al., 2023). Both mitochondrial decoupling and swelling have been observed in lethally frozen *C. costata* larvae. The existence of a colloidosmotic gradient in mitochondria of *D. melanogaster* S2 cells was demonstrated by permeabilizing the IMM with either the K^+^-specific ionophore valinomycin or the multicoductive pore-forming peptide alamethicin, both of which induced rapid mitochondrial swelling (Von Stockum et al., 2011) strikingly similar to what we observed in tissues from lethally frozen *C. costata*. IMM permeabilization followed by matrix swelling is a typical malformation induced by various pathologies, toxins or environmental stressors (Ghadially, 1988) and also accompanies apoptotic cell death in *D. melanogaster* S2 cells (Abdelwahid et al., 2007) and adult muscle cells (Klein et al., 2014). Taken together, there is little doubt that the mitochondrial swelling observed in lethally frozen *C. costata* larvae is caused by a loss of barrier function of the IMM. However, the cause of this freezing-induced permeability transition remains unclear.

### Hypothetical causes of permeability transition in IMM

There are at least two hypothetical explanations, which are not mutually exclusive: the occurrence of phase transitions in the lipid bilayer of the IMM; and the opening of a non-specific large pore in the IMM. The IMM may become porous due to the direct effects of low temperature and freeze dehydration on its lipid bilayer. As the ambient temperature gradually decreases, a specific temperature (*T*_m_) is reached at which the membrane phospholipids begin to transition from the liquid crystalline to the gel phase (Chapman, 1975). According to the theory developed by Quinn (1985), the non-bilayer-forming species, such as phosphatidylethanolamines (PEs), begin to transit earlier (at higher temperatures) than the bilayer-forming phosphatidylcholines. The membrane domains enriched in non-bilayer species are thus formed that are separated from the bilayer species domains. The loss of barrier function then occurs upon rewarming, when the domains of non-bilayer species tend to transition to the hexagonal phase, resulting in disruption of the bilayer structure and subsequent leakage of all solutes across the membrane in both directions. In addition, a decrease in membrane hydration associated with freezing stress can further worsen the situation, as the transition to the hexagonal phase becomes more likely with decreasing hydration of the phospholipid head groups (Quinn, 1985). Insect membranes generally contain a high proportion of non-bilayer forming lipid species (Koštál, 2010). For example, 51% of the phospholipid molecules in *C. costata* larval muscles are PEs and 63% of them carry at least one unsaturated fatty acyl chain (Koštál et al., 2003), making the PE molecules conical in shape and poorly bilayer packing. The composition of the *C. costata* IMM is currently unknown, but the IMM of other animals, including *D. melanogaster*, is known to have a very high content of non-bilayer forming species. In addition to approximately 30 mol% of PEs (Schenkel and Bakovic, 2014), the IMM contains approximately 15–20 mol% of cardiolipins (CLs) (Schlame et al., 2012; Gasanoff et al., 2021). The CL molecules, due to their highly conical molecular shape, are extremely prone to form non-bilayer structures such as inverted micelles and hexagonal phase under certain temperature and hydration conditions (Raison et al., 1971; Quinn, 1985; Hazel, 1995). Thus, the specific lipid composition appears to make the IMM relatively susceptible to loss of integrity due to direct effects of low temperature and low hydration on bilayer phase transitions.

Whether bilayer phase transitions actually occur in *mitochondrial* membranes of LD larvae exposed to freezing stress remains to be investigated. Nevertheless, we have already shown that *plasma* membrane integrity is compromised upon freezing stress in LD larvae but not in SDA larvae, where integrity is likely protected by at least two mechanisms: seasonal restructuring of lipid composition (Koštál et al., 2003); and stabilization by seasonally accumulated proline, trehalose and serum proteins (Grgac et al., 2022). Our unpublished observations confirmed that freezing-induced loss of plasma membrane integrity also occurs in other tissues of LD larvae, including muscle. The loss of plasma membrane integrity is likely to be followed by a massive influx of calcium from the extracellular space into the cytosol, which may have further deleterious consequences for mitochondria. The high cytosolic calcium can activate phospholipase C, which releases inositol phosphates (IPs) from plasma membrane phosphatidylinositols (Kadamur and Ross, 2013). The IPs are important signaling molecules that (among other things) open the calcium channels in the endoplasmic reticulum (ER) (Putney and Tomita, 2012; Kadamur and Ross, 2013), potentially closing the lethal feedback loop by releasing a secondary surge of calcium from the ER into the cytosol. More importantly, high cytosolic calcium is known to be a central player in the mammalian model of mitochondrial permeability transition, which is accompanied by characteristic mitochondrial swelling (Haworth and Hunter, 1979; Hunter and Haworth, 1979; Bernardi et al., 2023).

### Putative role of calcium in freezing stress-induced mitochondrial permeability transition

In both mammalian and insect models, high cytosolic calcium is inevitably and rapidly overloaded into the mitochondrial matrix (Bernardi et al., 2023), where it affects at least two targets related to the IMM permeability transition: cardiolipins and the permeability transition pore. First, the high innate tendency of CLs to form non-bilayer structures (see above) is known to be further stimulated by calcium (Verkleij, 1984). The formation of hexagonal non-bilayer structures induced by exogenous calcium was directly observed by freeze-fracture electron microscopy in isolated rat liver mitochondria (van Venetië and Verkleij, 1982). Second, in mammalian mitochondria, exogenous calcium concentrations higher than about 100 µM were shown to induce a permeability transition by opening a large non-selective channel in the IMM - the permeability transition pore (mPTP). The exact molecular identity of mPTP remains elusive despite decades of intensive efforts (Halestrap, 2009; Chinopoulos, 2018; Bernardi et al., 2023). The short opening of mPTP is thought to serve several regulatory purposes (e.g., reduction of calcium overload, reduction of ROS production). However, the prolonged mPTP openings cause mitochondrial swelling and rupture of the OMM, disrupting mitochondrial energy metabolism and ultimately leading to the induction of cell death pathways (Giorgio et al., 2018). The functional fly (*D. melanogaster*) homolog of mPTP has been characterized (Von Stockum et al., 2011). Although many functional similarities were found, it may differ from mammalian mPTP in that it does not form a large non-selective pore upon stimulation by calcium. No mitochondrial swelling was observed upon stimulation of *D. melanogaster* S2 cells permeabilized with digitonin and exposed to 40–100 µM calcium (Von Stockum et al., 2011). These observations led the authors to name the fly mPTP homologue mCRC—mitochondrial calcium release channel. The proposed function of mCRC under physiological conditions is to respond to relatively small and rapid pulses of calcium overload (e.g., in working muscle) by opening and releasing the excess calcium from the matrix (Von Stockum et al., 2011; Bernardi and von Stockum, 2012). A relative insensitivity of invertebrate mitochondria to doses of exogenous calcium between 100 and 1,000 µM has also been observed in the crustacean *Artemia franciscana* (Menze et al., 2005). However, it should be noted that extracellular calcium concentrations in the hemolymph of *D. melanogaster* larvae are as high as 3,600 µM (Krans et al., 2010). This leaves some room for speculation that much higher concentrations than those tested in *D. melanogaster* (100 µM) could be generated by calcium influx into the cytosol of cells in lethally frozen *C. costata* larvae, and that these concentrations would exert the effects described above either on the CL phase transition or on mCRC opening.

Follow-up studies are needed to assess the validity of the hypothesis of calcium mediation of the mitochondrial permeability transition in freeze-stressed insect cells. For example, in *C. costata* larvae, the experiments are ongoing to monitor the changes in cytosolic calcium levels (using calcium-sensitive probes) before and after freezing stress, while extracellular calcium levels and plasma membrane permeability are manipulated (by culturing tissues in media with differing calcium concentrations, and by permeabilization vs. stabilization of the plasma membrane by digitonin vs. cryoprotectants, respectively). It should also be noted that the influx of exogenous calcium into the cytosol plays a central role in the integrative model currently being constructed to explain chilling injury in chill-sensitive insects (such as the fly *D. melanogaster* or the migratory locust, *Locusta migratoria*) (MacMillan, 2019). Chilling injury is caused by relatively short exposures to mild temperatures, typically above zero. In the *chilling* injury model, the deleterious cascade begins with a change in plasma membrane *function*: the ion motive activity of Na^+^/K^+^ ATPase decreases with decreasing ambient temperature and cannot keep up with the backward flow of ions. This leads to a loss of ionic and osmotic balance across the body epithelia, resulting in systemic cell membrane depolarization (Koštál et al., 2004; MacMillan et al., 2015; Overgaard and MacMillan, 2017), which in turn causes the opening of the calcium voltage-dependent channels (Bayley et al., 2018; Bayley et al., 2019; Bayley et al., 2020). In the hypothetical *freezing* injury model outlined here, the deleterious cascade would begin with the loss of plasma membrane *structure*. Freezing stress is hypothesized to be associated with the formation of non-bilayer structures in the plasma membrane that are leaky not only to calcium but also to any large and charged molecule (such as Trypan blue).

## Conclusion

In conclusion, we have shown that lethal freezing stress in *C. costata* larvae is closely associated with mitochondrial swelling. Since muscle OXPHOS proteins remained at least partially functional after lethal freezing stress, we hypothesized that the permeability transition in the muscle IMM lipid bilayer is primarily responsible for the loss of matrix volume control and subsequent mitochondrial swelling. In the search for plausible mechanistic explanations for the cause of the IMM permeability transition, we outlined two mutually non-exclusive hypotheses, which postulate that: 1) the direct effects of cold and freeze dehydration on IMM lipid bilayer phase transitions may result in the formation of non-bilayer structural defects that render the IMM leaky, and 2) IMM permeabilization is induced by calcium overload of the mitochondrial matrix following the surge of extracellular calcium into the cytosol resulting from loss of plasma membrane integrity. We suggest that further investigation in these two directions may help to understand the mechanistic nature of freeze-induced injury in freeze-sensitive cells and animals.

## Data Availability

The original contributions presented in the study are included in the article/Supplementary Material, further inquiries can be directed to the corresponding author.
