# Supplementary material for: Extracellular freezing induces a permeability transition in the inner membrane of muscle mitochondria of freeze-sensitive but not freeze-tolerant Chymomyza costata larvae
Source: Front Physiol. 2024 Feb 7;15:1358190. doi: 10.3389/fphys.2024.1358190 (PMC10880108; doi:10.3389/fphys.2024.1358190)
Supplement: Supplementary file 1 [file DataSheet1.PDF]

Extracellular freezing induces a permeability transition in the inner membrane of muscle mitochondria of freeze-sensitive but not freeze-tolerant *Chymomyza costata* larvae.

Tomáš Štětina[1], Vladimír Koštál[1]

[1] Institute of Entomology, Biology Centre of the Czech Academy of Sciences, České Budějovice, Czech Republic

Supplementary material

Supplementary Figures:

Figure S1: Classification of morphology of muscle mitochondria of the larvae of *Chymomyza costata*
Figure S2: Methods
Figure S3: Examples of muscle mitochondria in control unfrozen larvae of *Chymomyza costata*
Figure S4: Examples of muscle mitochondria in larvae of *Chymomyza costata* frozen to -30°C
Figure S5: Examples of original traces of oxygen concentration and oxygen flux taken by Oxygraph-2K for activity of complex I
Figure S6: The respiration rates linked to activity of complex II were significantly reduced after the freezing stress in the muscles of freeze-sensitive larvae of *Chymomyza costata*
Figure S7: Examples of original traces of oxygen concentration and oxygen flux taken by Oxygraph-2K for activity of complex II

Supplementary Tables

raw data used for statistical analyses of the results presented in Figures:

Figure 1: Activity of citrate synthase in larval muscles
Figure 2: Scoring of mitochondrial morphology
Figure 3: Activity of electron transfer chain in larval muscles (Alamar Blue assay)
Figure 4: Basal respiration rates and substrate contribution ratios (Oxygraph 2K)
Figure 5: Oxygen consumption data, Complex I (Oxygraph 2K)
Figure 6: Coupling efficiency (Oxygraph 2K)
Figure S6: Oxygen consumption data, Complex II (Oxygraph 2K)

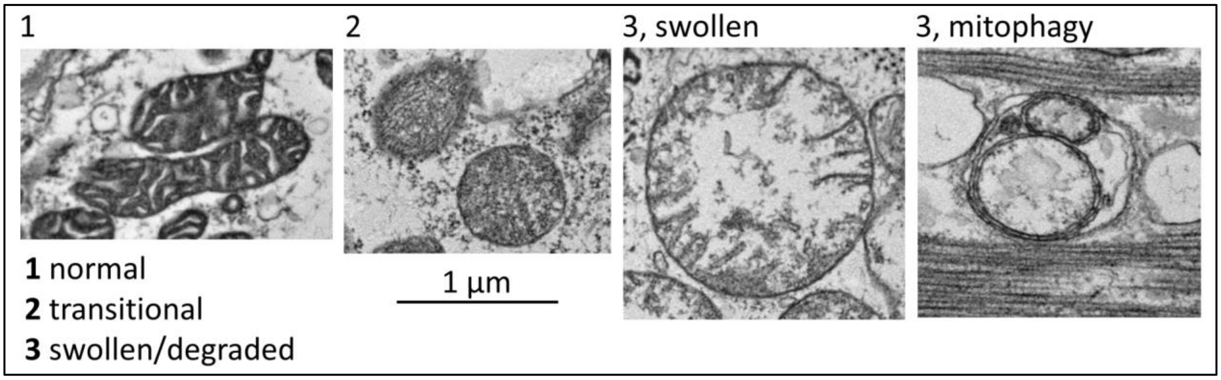

Figure S1: Classification of morphology of muscle mitochondria of the larvae of *Chymomyza costata*.

The transmission electron micrographs were taken at a magnification of 25,000x using the microscope JEOL JEM - 1010 1 (JEOL, Tokio, Japan). Three morphological classes of mitochondria were distinguished: (1) normal; such mitochondria are abundantly present in the muscle of LD and SDA control larvae (the sizes and shapes are highly variable; many normal mitochondria are elongated or rod-like reaching total length of up to ca. 3 µm; on transversal section, the diameter does not exceed ca. 1 µm); (2) transitional (slightly enlarged, rounded, cristae invisible, matrix is full of electron-dense material; it may also be a section of swollen rounded mitochondria close to its periphery); and (3) swollen; such mitochondria are abundantly present in lethally frozen LD larvae (the mitochondria are rounded, clearly enlarged, often with diameter much higher than µm 1; the cristae are displaced to the periphery or invisible, the matrix is diluted (i.e. it does not contain electron dense material); the OMM may be discontinuous). In addition, the mitochondria undergoing degradation (mitophagy) in autophagosomes were occasionally seen in all experimental variants and those were also scored as (3).

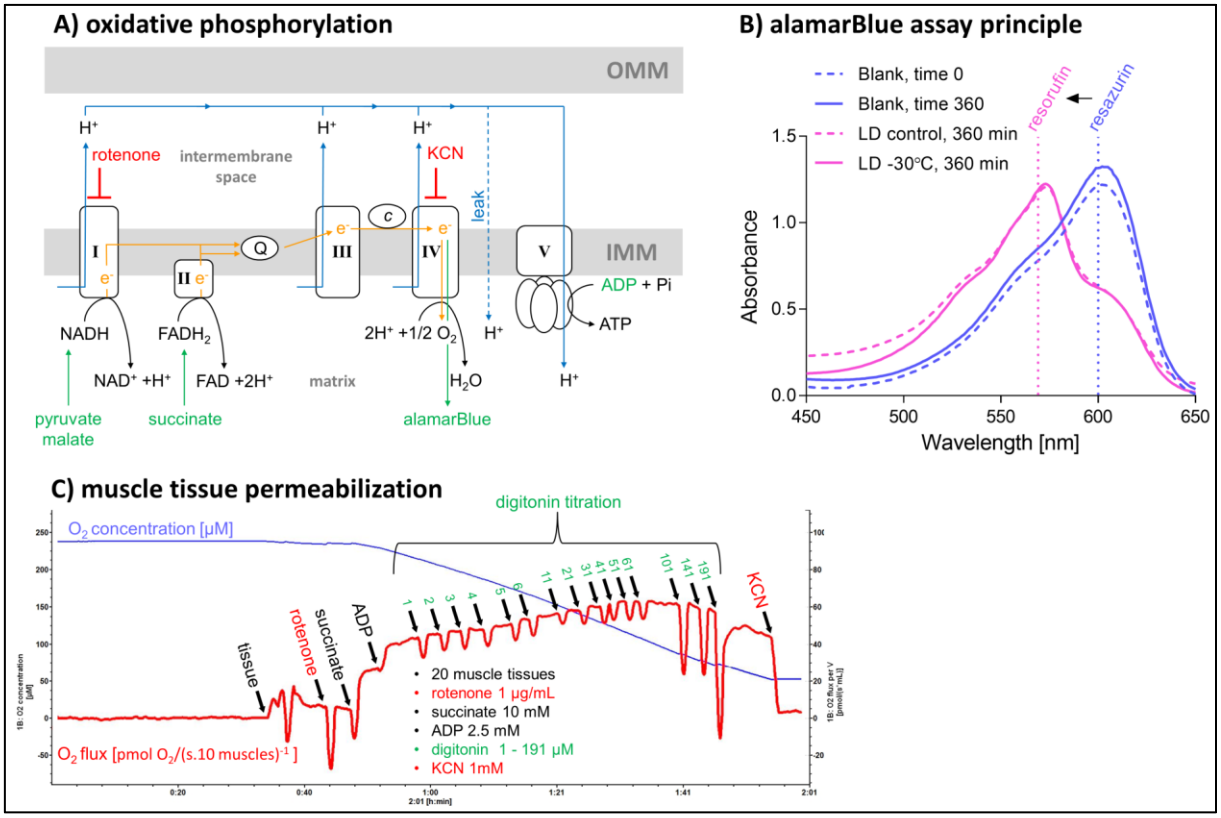

Figure S2: Methods.

A) Scheme of mitochondrial electron transfer chain and oxidative phosphorylation (oxphos) system. IMM, inner mitochondrial membrane; OMM, outer mitochodnrial membrane; complex I, NADH ubiquinone oxidoreductase; complex II, succinate dehydrogenase; complex III, cytochrome *c* reductase; complex IV, cytochrome *c* oxidase; complex V, ATP synthase; Q, ubiquinone; *c*, cytochrome c. The orange and blue arrows show the flows of electrons and protons, respectively, through the system. The inhibitory effects of rotenone and KCN are shown as red line segments. Green font is used to show respiratory substrates and reagents that were used in assays.

B) The shift in absorbance of the alamarBlue assay with time of incubation.

Four examples of spectra are shown: (i) blank (no tissue), taken at time 0 (right upon the addition of 10 µL of alamarBlue reagent and mixing); (ii) blank taken at time 360 min; (iii) sample of 5 muscle tissues dissected from unfrozen control LD larvae at time 360 min; (iv) sample of 5 muscle tissues dissected from lethally frozen (to -30°C) LD larvae at time 360 min (note: at time 0, the assays containing tissue have the same color as blank). It is obvious that samples of control and lethally frozen larvae behaved almost equally – the resazurin was transformed to resorufin within 360 min of incubation, which indicates similar metabolic activity in both control and lethally frozen samples.

C) Digitonin titration for permeabilization of LD muscle tissue.

Traces of oxygen concentration (blue) and flux (red) from Oxygraph-2K respirometer (Oroboros). The muscle tissues were dissected from 20 *C. costata* larvae (LD Control, unfrozen), pooled and transferred to 2 mL of respiration buffer in the respirometer chamber. Each injection is represented with an arrow indicating the compound injected and the final concentration of each compound in the medium. The diagram shows the effect of permeabilization of LD muscle cell membranes with gradually increasing concentrations of digitonin. The titration indicated that 55 µM concentration of digitonin is optimal for the LD tissue. Analogous titration was performed in the SDA tissue and indicated that 30 µM digitonin is the optimal concentration for it.

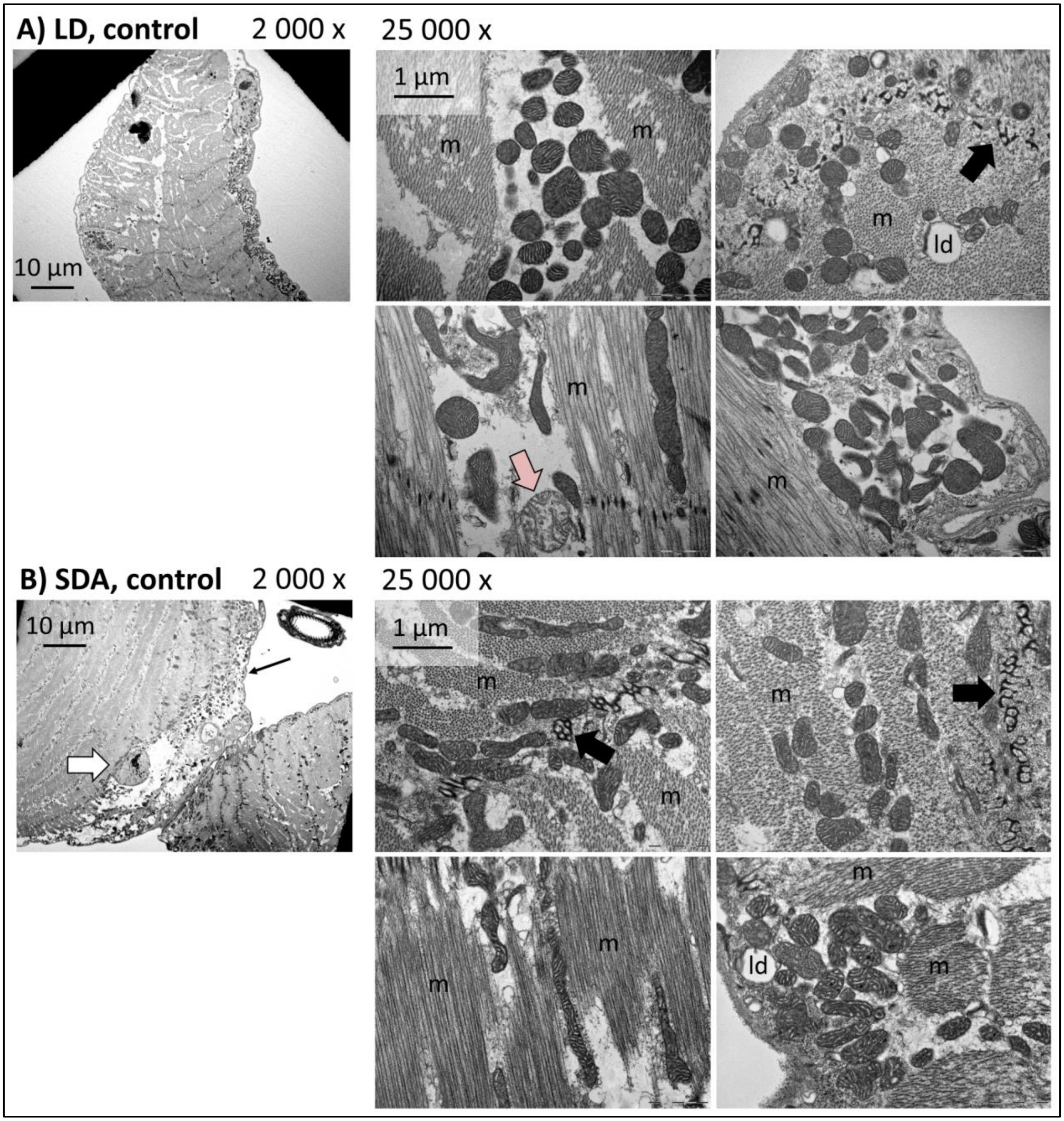

Figure S3: Examples of muscle mitochondria in control unfrozen larvae of *Chymomyza costata.*

The transmission electron micrographs taken at a magnification of 2,000x show the overall structure of muscle cells (large white arrows point to nuclei) with large populations of mitochondria close to plasma membrane (small black arrows) and other mitochondria interspersed among myofibrils. The micrographs taken at a magnification of 25,000x show variability in mitochondrial shapes. Most mitochondria, however can be scored as normal (1); pink arrow points to a single mitochondrion classified as swollen (3). Large black arrows point to sarcoplasmic reticulum; ld, lipid droplet; m, myofibrils. A gallery containing all micrographs and scores is available in figshare:
https://figshare.com/articles/figure/Gallery_of_mitochondria_pdf/24961422

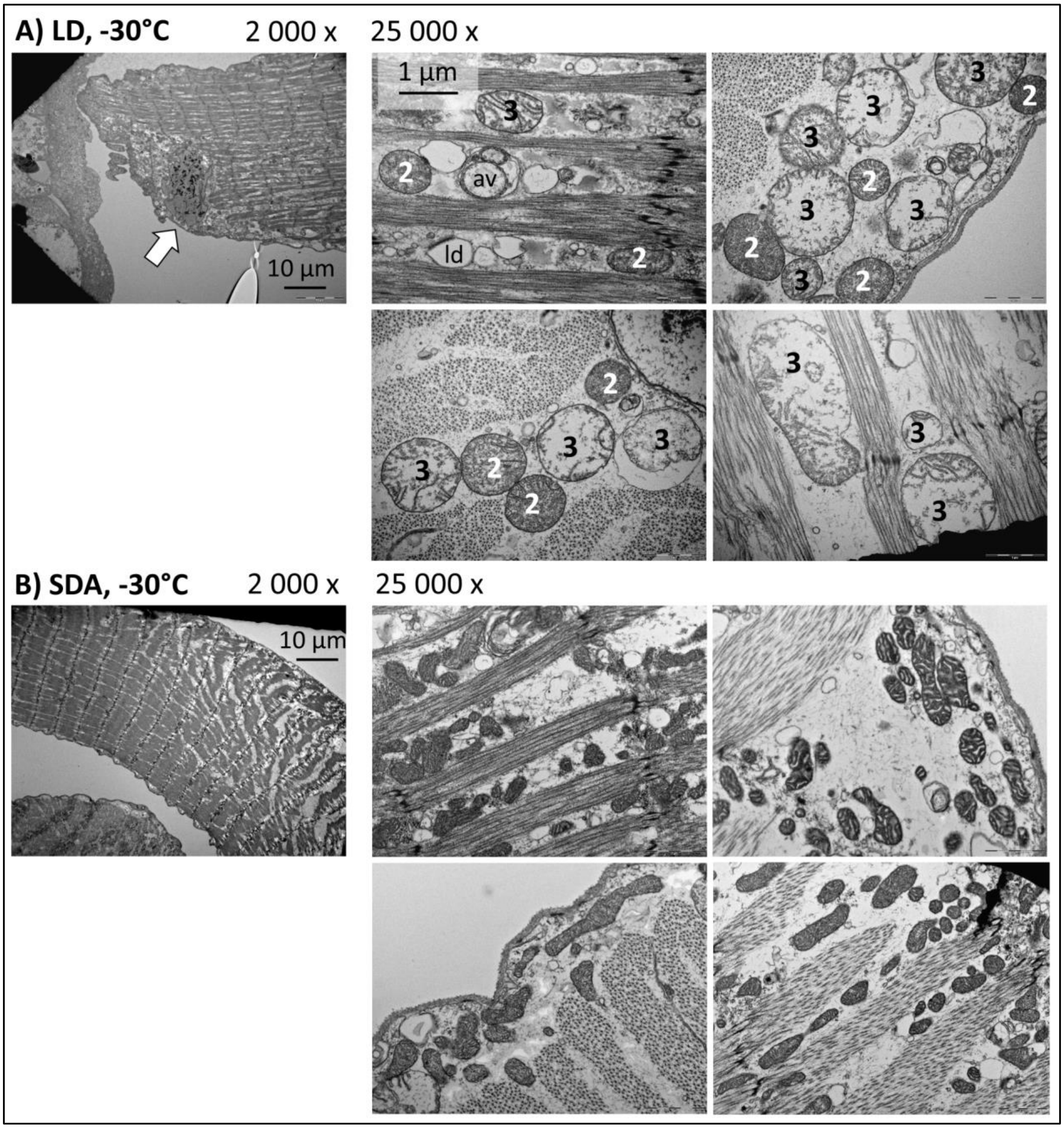

Figure S4: Examples of muscle mitochondria in larvae of *Chymomyza costata* frozen to -30°C.

The transmission electron micrographs taken at a magnification of 2,000x show that the overall structure of muscle cells does not change upon freezing (large white arrow points to nucleus). The micrographs taken at a magnification of 25,000x show that most mitochondria of LD larvae (**A**) deviate from normal morphology and are scored as either transitional (2) or swollen (3) (av, autophagic vacuole; ld, lipid droplet). Most mitochondria of SDA larvae (**B**), however, can be scored as normal (1). A gallery containing all micrographs and scores is available in figshare: https://figshare.com/articles/figure/Gallery_of_mitochondria_pdf/24961422

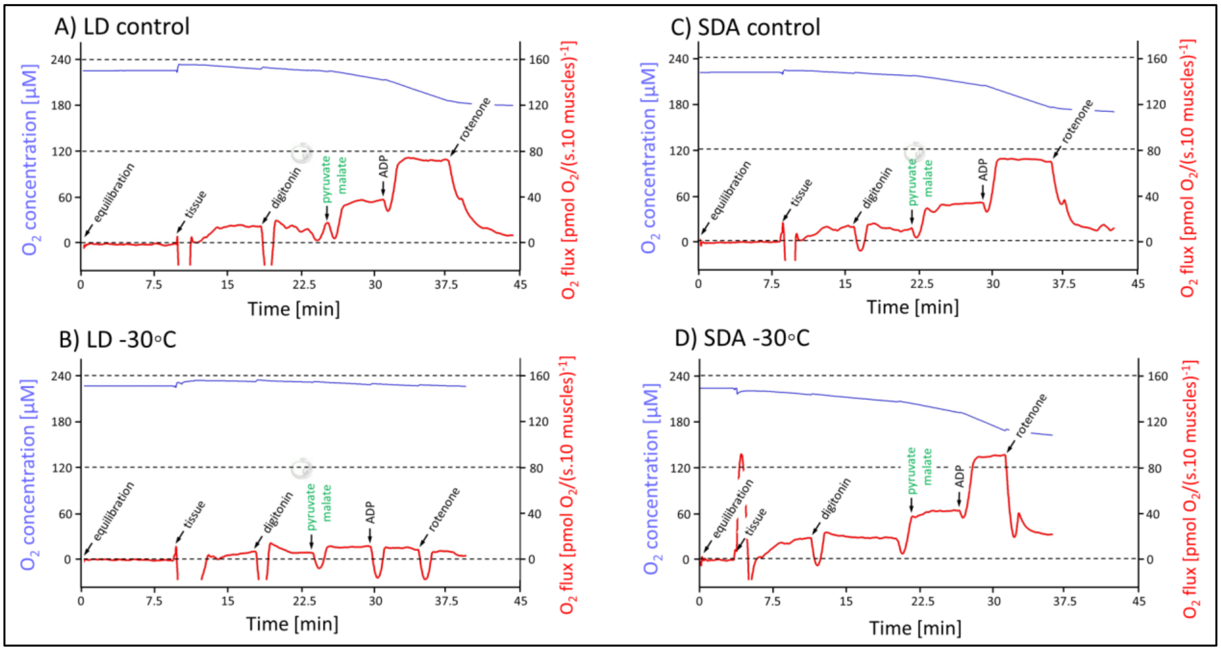

Figure S5: Examples of original traces of oxygen concentration (blue lines) and oxygen flux (red lines) taken by Oxygraph-2K for activity of complex I.

The activity of complex I in digitonin-permeabilized larval muscle tissue (20 tissue pooled per respiration chamber, corresponding to 10 tissue per mL of respiration buffer) was supported by specific substrates pyruvate and malate (to obtain substrate-stimulated oxygen flux, SS), followed by ADP (to obtain OXPHOS flux), and finally rotenone to inhibit the complex I activity. The arrows show exact moments of the injections of different additives.

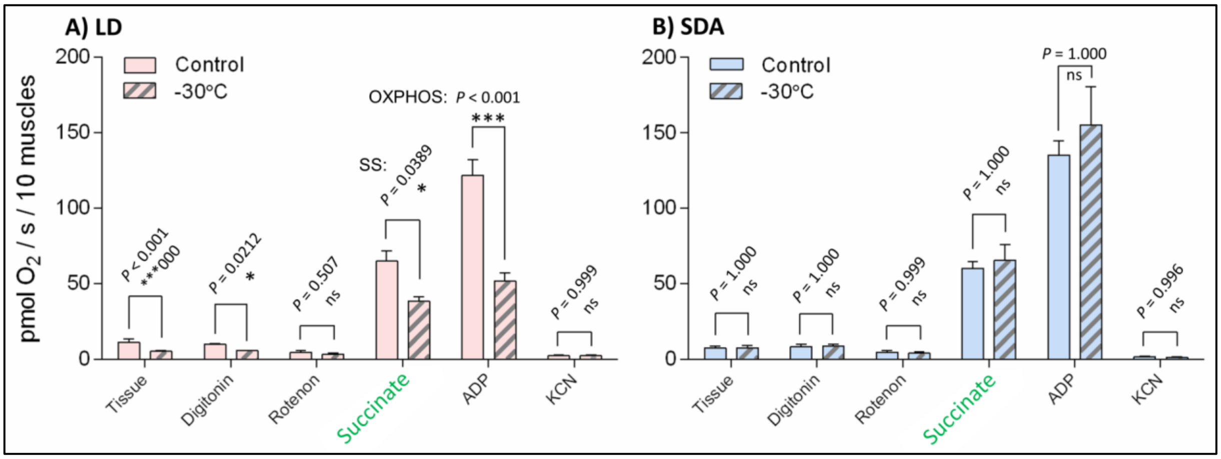

Figure S6: The respiration rates linked to activity of complex II were significantly reduced after the freezing stress in the muscles of freeze-sensitive larvae of *Chymomyza costata*. The freeze-sensitive (LD) and freeze-tolerant (SDA) larvae were exposed to slow inoculative freezing to -30°C. The muscle tissues were dissected right after the freezing stress (or taken from control-unfrozen larvae), permeabilized using digitonin, and the the oxygen consumption rates were measured using Oxygraph-2K respirometer after adding succinate – specific substrate for complex II (substrate-stimulated oxygen flux, SS), followed by ADP (OXPHOS state, activity of electron transfer chain coupled to ATP synthase), followed by KCN – specific inhibitor of complex IV (see Supplementary Figure S2A). Each column shows mean ± SD (n = 3, each replicate contained a pool of 20 dissected larval muscle tissues). The differences between frozen and control variants (see clamps) were statistically tested using multiple ANOVA models (checked for nested effects). Stars represent statistical differences (*P <0.05; **P <0.01; ***P <0.001; ns, not significant). In case of LD larvae (A), the log-transformed oxygen consumption data were used. See Supplementary Figure S7 for examples of original oxygen flux traces taken by Oxygraph-2K).

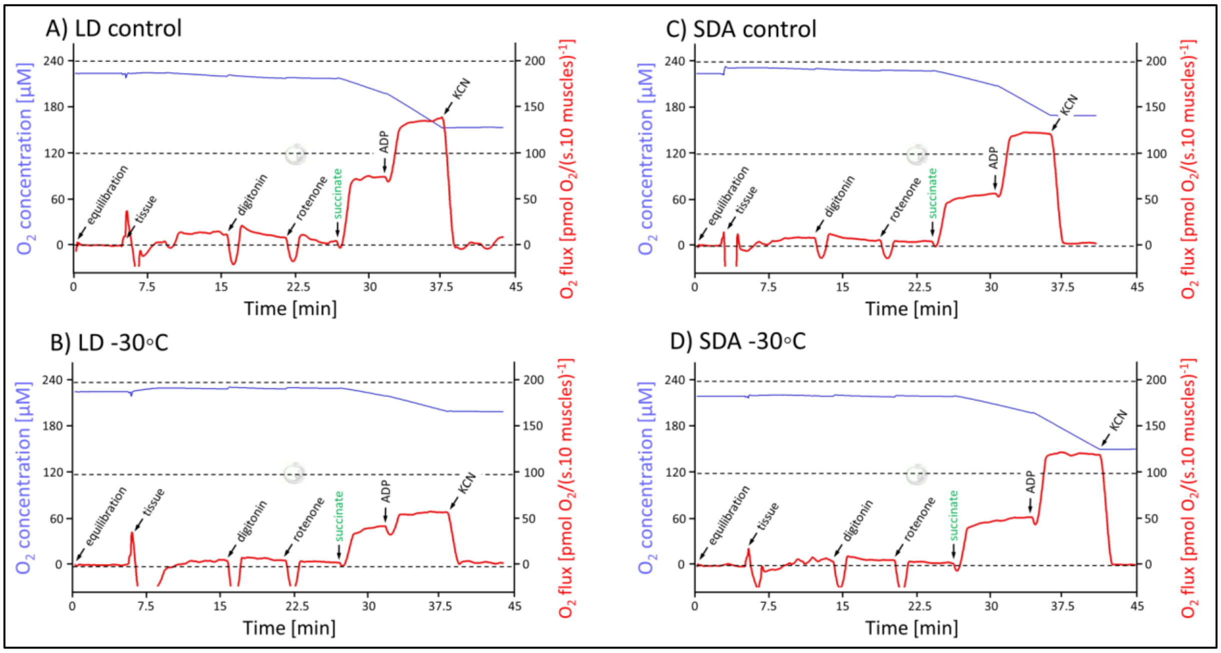

Figure S7: Examples of original traces of oxygen concentration (blue lines) and oxygen flux (red lines) taken by Oxygraph-2K for activity of complex II.

The activity of complex II in digitonin-permeabilized larval muscle tissue (20 tissue pooled per respiration chamber, corresponding to 10 tissue per mL of respiration buffer) was supported by specific substrate succinate (to obtain substrate-stimulated oxygen flux, SS), followed by ADP (to obtain OXPHOS flux), and finally KCN to inhibit the complex IV activity. The arrows show exact moments of the injections of different additives.

Raw data for Fig. 1
Activity of citrate synthase in larval muscles

µmol DNTB oxidized min^{-1} mg^{-1} muscle total protein

replicate	LD control	SDA control
1	443,2	403,9
2	508,0	412,4
3	510,7	393,5
mean:	**487,3**	**403,3**
SD:	31,2	7,7

each replicate represents response measured in the pool of muscle tissues dissected from 10 larvae

Raw data for Fig. 2
Scoring of mitochondrial morphology

LD Control - counts

replicate	normal	transitional	swollen	TOTAL
1	389	14	14	**417**
2	311	6	26	**343**
3	551	2	26	**579**
4	204	0	3	**207**
5	333	1	60	**394**
				**1 940**

LD Control - percentages

	normal	transitional	swollen	TOTAL
	93,3	3,4	3,4	**100**
	90,7	1,7	7,6	**100**
	95,2	0,3	4,5	**100**
	98,6	0,0	1,4	**100**
	84,5	0,3	15,2	**100**
mean:	**92,4**	**1,1**	**6,4**	
SD:	4,7	1,3	4,8	

LD -30*C - counts

replicate	normal	transitional	swollen	TOTAL
1	53	113	221	**387**
2	14	88	94	**196**
3	15	56	170	**241**
4	19	50	113	**182**
5	91	76	177	**344**
				**1 350**

LD -30*C - percentages

	normal	transitional	swollen	TOTAL
	13,7	29,2	57,1	**100**
	7,1	44,9	48,0	**100**
	6,2	23,2	70,5	**100**
	10,4	27,5	62,1	**100**
	26,5	22,1	51,5	**100**
mean:	**12,8**	**29,4**	**57,8**	
SD:	7,3	8,2	8,0	

SDA Control - counts

replicate	normal	transitional	swollen	TOTAL
1	227	2	9	**238**
2	252	6	16	**274**
3	242	0	10	**252**
4	385	15	8	**408**
5	435	3	20	**458**
				**1 630**

SDA Control - percentages

	normal	transitional	swollen	TOTAL
	95,4	0,8	3,8	**100**
	92,0	2,2	5,8	**100**
	96,0	0,0	4,0	**100**
	94,4	3,7	2,0	**100**
	95,0	0,7	4,4	**100**
mean:	**94,5**	**1,5**	**4,0**	
SD:	1,4	1,3	1,2	

SDA -30*C - counts

replicate	normal	transitional	swollen	TOTAL
1	143	43	26	**212**
2	205	18	24	**247**
3	291	4	30	**325**
4	252	16	42	**310**
5	151	18	108	**277**
				**1 371**

SDA -30*C - percentages

	normal	transitional	swollen	TOTAL
	67,5	20,3	12,3	**100**
	83,0	7,3	9,7	**100**
	89,5	1,2	9,2	**100**
	81,3	5,2	13,5	**100**
	54,5	6,5	39,0	**100**
mean:	**75,2**	**8,1**	**16,7**	
SD:	12,6	6,4	11,2	

Raw data for Fig. 3
Metabolic activity of larval muscle cells (Alamar Blue assay)

Absorbances measured at time 0 min

treatment	replicate	Abs 570	Abs 600	Abs 600 / Abs 570
Blank time 0	1	0,761	1,173	1,54
	2	0,824	1,259	1,53
	3	0,727	1,166	1,60
			mean:	**1,56**
			SD:	0,03

each replicate represents response measured in the pool of muscle tissues dissected from 5 larvae

Absorbances measured after incubation for 360 min

treatment	replicate	Abs 570	Abs 600	Abs 600 / Abs 570
Blank time 360	1	0,846	1,281	1,51
	2	0,726	1,152	1,59
	3	0,812	1,126	1,39
			mean:	**1,50**
			SD:	0,08
LD +65*C	1	0,876	1,197	1,37
	2	0,975	1,327	1,36
	3	0,701	1,011	1,44
			mean:	**1,39**
			SD:	0,04
LD +45*C	1	1,627	0,340	0,21
	2	1,300	0,322	0,25
	3	1,531	0,310	0,20
			mean:	**0,22**
			SD:	0,02
LD Control	1	1,395	0,299	0,21
	2	1,248	0,358	0,29
	3	1,183	0,629	0,53
			mean:	**0,34**
			SD:	0,14
LD -30*C	1	1,209	0,620	0,51
	2	1,226	0,876	0,71
	3	1,249	0,689	0,55
			mean:	**0,59**
			SD:	0,09
SDA Control	1	1,322	0,328	0,25
	2	1,239	0,319	0,26
	3	1,205	0,319	0,26
			mean:	**0,26**
			SD:	0,01
SDA -30*C	1	1,504	0,428	0,28
	2	1,531	0,419	0,27
	3	1,410	0,398	0,28
			mean:	**0,28**
			SD:	0,00

Raw data for Fig. 4
Basal respiration rates and substrate contribution ratios (Oxygraph 2K)

Basal oxygen consumption in digiton-permeabilized larval muscles
pmol O_2 / s / 1 mL (10 muscles) (assay volume = 2 mL)

assay	replicate	LD Control	LD -30*C	SDA Control	SDA -30*C
Complex I	1	12	7,5	12	19
	2	13	6,5	12	14,5
	3	9,5	5,5	15,5	17,5
Complex II	4	9,5	5,5	7,5	6,5
	5	10,5	5,5	7	10
	6	10	6	10,5	9
	mean:	**10,75**	**6,08**	**10,75**	**12,75**
	SD:	1,31	0,73	2,90	4,57

each replicate represents response measured in the pool of muscle tissues dissected from 20 larvae

Substrate contribution ratio of pyruvate and malate
(SS – basal)/basal

assay	replicate	LD Control	LD -30*C	SDA Control	SDA -30*C
Complex I	1	1,92	2,13	1,88	1,26
	2	1,88	0,77	2,42	1,07
	3	2,47	1,91	1,35	1,11
	mean:	**2,09**	**1,60**	**1,88**	**1,15**
	SD:	0,27	0,60	0,43	0,08

Substrate contribution ratio of succinate
(SS – basal)/basal

assay	replicate	LD Control	LD -30*C	SDA Control	SDA -30*C
Complex II	1	13,80	15,20	17,86	16,33
	2	10,45	7,89	11,33	18,14
	3	18,00	10,33	8,92	13,09
	mean:	**14,08**	**11,14**	**12,70**	**15,86**
	SD:	3,09	3,04	3,78	2,09

Raw data for Fig. 5
Oxygen consumption data, Complex I (Oxygraph 2K)

Basal oxygen consumption prior to digiton addition
pmol O_2 / s / 1 mL (10 muscles) (assay volume = 2 mL)

assay	replicate	LD Control	LD -30*C	SDA Control	SDA -30*C
Tissue	1	13,00	7,50	13,00	18,50
	2	15,50	6,00	11,00	13,50
	3	11,50	6,00	14,50	17,00
	mean:	**13,33**	**6,50**	**12,83**	**16,33**
	SD:	1,65	0,71	1,43	2,09

each replicate represents response measured in the pool of muscle tissues dissected from 20 larvae

Basal oxygen consumption in digiton-permeabilized larval muscles
pmol O_2 / s / 1 mL (10 muscles) (assay volume = 2 mL)

assay	replicate	LD Control	LD -30*C	SDA Control	SDA -30*C
Digitonin	1	12,00	7,50	12,00	19,00
	2	13,00	6,50	12,00	14,50
	3	9,50	5,50	15,50	17,50
	mean:	**11,50**	**6,50**	**13,17**	**17,00**
	SD:	1,47	0,82	1,65	1,87

Substrate-stimulated (SS) oxygen consumption after adding substrates (pyruvate and malate)
pmol O_2 / s / 1 mL (10 muscles) (assay volume = 2 mL)

assay	replicate	LD Control	LD -30*C	SDA Control	SDA -30*C
SS	1	35,00	23,50	34,50	43,00
	2	37,50	11,50	41,00	30,00
	3	33,00	16,00	36,50	37,00
	mean:	**35,17**	**17,00**	**37,33**	**36,67**
	SD:	1,84	4,95	2,72	5,31

OXPHOS oxygen consumption after adding ADP
pmol O_2 / s / 1 mL (10 muscles) (assay volume = 2 mL)

assay	replicate	LD Control	LD -30*C	SDA Control	SDA -30*C
OXPHOS	1	66,00	27,00	73,00	91,00
	2	73,50	10,50	84,00	65,00
	3	69,00	19,50	73,00	71,50
	mean:	**69,50**	**19,00**	**76,67**	**75,83**
	SD:	3,08	6,75	5,19	11,05

Oxygen consumption after adding rotenone
pmol O_2 / s / 1 mL (10 muscles) (assay volume = 2 mL)

assay	replicate	LD Control	LD -30*C	SDA Control	SDA -30*C
KCN	1	7,00	5,00	11,00	22,00
	2	7,50	3,50	9,50	17,50
	3	7,00	5,00	18,50	20,50
	mean:	**7,17**	**4,50**	**13,00**	**20,00**
	SD:	0,24	0,71	3,94	1,87

Raw data for Fig. 6
Coupling efficiency (Oxygraph 2K)

Coupling efficiency
1 – (SS/OXPHOS)

assay	replicate	LD Control	LD -30*C	SDA Control	SDA -30*C
Complex I	1	0,47	0,13	0,53	0,53
	2	0,49	-0,10	0,51	0,54
	3	0,52	0,18	0,50	0,48
	mean:	**0,49**	**0,07**	**0,51**	**0,52**
	SD:	0,02	0,12	0,01	0,02

each replicate represents response measured in the pool of muscle tissues dissected from 20 larvae

assay	replicate	LD Control	LD -30*C	SDA Control	SDA -30*C
Complex II	1	0,46	0,28	0,54	0,57
	2	0,47	0,26	0,55	0,59
	3	0,48	0,24	0,58	0,58
	mean:	**0,47**	**0,26**	**0,55**	**0,58**
	SD:	0,01	0,02	0,02	0,01

Raw data for Fig. S6
Oxygen consumption data, Complex II (Oxygraph 2K)

Basal oxygen consumption prior to digiton addition
pmol O_2 / s / 1 mL (10 muscles)   (assay volume = 2 mL)

assay	replicate	LD Control	LD -30*C	SDA Control	SDA -30*C
Tissue	1	13,50	4,50	5,50	5,50
	2	11,50	6,00	8,50	7,50
	3	9,00	5,00	8,50	9,50
	mean:	**11,33**	**5,17**	**7,50**	**7,50**
	SD:	1,84	0,62	1,41	1,63

each replicate represents response measured in the pool of muscle tissues dissected from 20 larvae

Basal oxygen consumption in digiton-permeabilized larval muscles
pmol O_2 / s / 1 mL (10 muscles)   (assay volume = 2 mL)

assay	replicate	LD Control	LD -30*C	SDA Control	SDA -30*C
Digitonin	1	9,50	5,50	7,50	6,50
	2	10,50	5,50	7,00	10,00
	3	10,00	6,00	10,50	9,00
	mean:	**10,00**	**5,67**	**8,33**	**8,50**
	SD:	0,41	0,24	1,55	1,47

Oxygen consumption after adding rotenone
pmol O_2 / s / 1 mL (10 muscles)   (assay volume = 2 mL)

assay	replicate	LD Control	LD -30*C	SDA Control	SDA -30*C
Rotenone	1	5,00	2,50	3,50	3,00
	2	5,50	4,50	4,50	3,50
	3	3,00	3,00	6,00	5,50
	mean:	**4,50**	**3,33**	**4,67**	**4,00**
	SD:	1,08	0,85	1,03	1,08

Substrate-stimulated (SS) oxygen consumption after adding substrate (succinate)
pmol O_2 / s / 1 mL (10 muscles)   (assay volume = 2 mL)

assay	replicate	LD Control	LD -30*C	SDA Control	SDA -30*C
SS	1	74,00	40,50	66,00	52,00
	2	63,00	40,00	55,50	67,00
	3	57,00	34,00	59,50	77,50
	mean:	**64,67**	**38,17**	**60,33**	**65,50**
	SD:	7,04	2,95	4,33	10,46

OXPHOS oxygen consumption after adding ADP
pmol O_2 / s / 1 mL (10 muscles)   (assay volume = 2 mL)

assay	replicate	LD Control	LD -30*C	SDA Control	SDA -30*C
OXPHOS	1	136,00	56,50	143,50	121,00
	2	118,00	54,00	122,00	162,00
	3	110,50	44,50	140,50	182,50
	mean:	**121,50**	**51,67**	**135,33**	**155,17**
	SD:	10,70	5,17	9,51	25,57

Oxygen consumption after adding KCN
pmol O_2 / s / 1 mL (10 muscles)   (assay volume = 2 mL)

assay	replicate	LD Control	LD -30*C	SDA Control	SDA -30*C
KCN	1	2,00	2,00	1,50	1,00
	2	2,00	3,00	2,50	2,00
	3	3,00	1,50	0,50	0,50
	mean:	**2,33**	**2,17**	**1,50**	**1,17**
	SD:	0,47	0,62	0,82	0,62
